# Comparing the diagnostic performance of QuantiFERON-TB Gold Plus with QFT-GIT, T-SPOT.TB and TST: a systematic review and meta-analysis

**DOI:** 10.1186/s12879-023-08008-2

**Published:** 2023-01-20

**Authors:** Yu Zhang, Guozhong Zhou, Wei Shi, Weili Shi, Meijun Hu, Defu Kong, Rong Long, Jian He, Nan Chen

**Affiliations:** 1 Department of Endocrinology, Anning First People’s Hospital Affiliated to Kunming University of Science and Technology, Kunming, 650302 Yunnan China; 2 Department of Science and Research, Anning First People’s Hospital Affiliated to Kunming University of Science and Technology, Kunming, 650302 Yunnan China; 3 Department of Pulmonary and Critical Care Medicine, Anning First People’s Hospital Affiliated to Kunming University of Science and Technology, Kunming, 650302 Yunnan China

**Keywords:** Latent tuberculosis infection, QuantiFERON-TB Gold plus, Sensitivity, Specificity, Positive rate

## Abstract

**Background:**

QuantiFERON-TB Gold Plus (QFT-Plus) is an important test that has emerged in recent years for detecting TB infection. We conducted a review to compare the sensitivity, specificity and positive rate of QFT-Plus with that of QuantiFERON-TB Gold In-Tube (QFT-GIT), T-cell spot of tuberculosis assay (T-SPOT.TB) and Tuberculin test (TST).

**Methods:**

PubMed and Embase were searched, without language restrictions, from 1 January 2015 to 31 March 2022 using “*Mycobacterium tuberculosis* Infections” and “QuantiFERON-TB-Plus” as search phrases. We estimated the sensitivity from studies of patients with active tuberculosis, specificity from studies of populations with very low risk of TB exposure, and positive rate from studies of high-risk populations. The methodological quality of the eligible studies was assessed, and a random-effects model meta-analysis was used to determine the risk difference (RD). We assessed the pooled rate by using a random-effects model. This study was registered in PROSPERO (CRD 42021267432).

**Results:**

Of 3996 studies, 83 were eligible for full-text screening and 41 were included in the meta-analysis. In patients with active TB, the sensitivity of QFT-Plus was compared to that of QFT-GIT and T-SPOT.TB, respectively, and no statistically differences were found. In populations with a very low risk of TB exposure, the specificity of QFT-Plus was compared with that of QFT-GTI and T-SPOT.TB, respectively, and no statistically differences were found. Two studies were eligible to compare the specificity of the QFT-Plus test with that of the TST test, and the pooled RD was 0.12 (95% CI 0.02 to 0.22). In high-risk populations, 18 studies were eligible to compare the positive rate of the QFT-Plus test with that of the QFT-GIT test, and the pooled RD was 0.02 (95% CI 0.01 to 0.03). The positive rate of QFT-Plus was compared with that of T-SPOT.TB and TST groups, and no statistically differences were found.

**Conclusions:**

The diagnostic performance of QFT-Plus was similar to that of QFT-GIT and T-SPOT.TB, but was slightly more specific than TST.

**Supplementary Information:**

The online version contains supplementary material available at 10.1186/s12879-023-08008-2.

## Background

Tuberculosis is a chronic infectious disease caused by *Mycobacterium tuberculosis*. Approximately one-quarter of the world’s population is currently infected with *Mycobacterium tuberculosis*, most of whom also have latent TB infections [1, 2]. With the World Health Organization’s (WHO) goal of eliminating TB by 2050, it is particularly important to accurately diagnose people with TB [3–5].

For many years, the common method used to diagnose TB was the tuberculin test (TST), which is inexpensive and easy to perform, but is prone to false-negative results in immunosuppressed patients and false-positive results in patients following Bacillus Calmette-Guérin (BCG) vaccination or those infected with non-tuberculous mycobacteria (NTM) [6, 7]. Subsequently, two methods for detecting TB emerged: QuantiFERON-TB Gold In Tube (QFT-GIT) and T-SPOT.TB. However, one study found no significant difference between TB detection using T-SPOT.TB compared with TST in an immunocompromised population, and another study found no significant difference between QFT-GIT and TST in children [6, 8].

In 2015, Qiagen launched a new generation of Interferon-γ release assays that include an additional TB2 tube called QTF-Plus. The manufacturer claims the newly released assays are more sensitive than previous versions because they enable the stimulation of CD4+ and CD8+ T cells, which results in the production of IFN-γ [9–11]. Nevertheless, previous studies have shown no significant improvement in the performance of QFT-Plus compared to QFT-GIT [12, 13].

We conducted this review to compare the sensitivity in active TB patients, specificity in populations with a very low risk of TB exposure, and a positive rate in high-risk populations of QFT-Plus with QFT-GIT, T-SPOT.TB and TST to evaluate the performance of QFT-Plus with QFT-GIT, T-SPOT.TB and TST assays.

## Methods

Preferred Reporting Items for Systematic Reviews and Meta-Analyses (PRISMA) guidelines were adhered to when conducting the meta-analysis (Additional file 1: Table S1), and the study was registered in PROSPERO (CRD42021267432).

### Data sources and search strategy

We searched PUBMED and EMBASE of Systematic Reviews for studies published from 1 January 2015 to 31 March 2022 with no language restrictions. The starting date of the search was based on the fact that QFT-Plus was first released by Qiagen in 2015. Search keywords included “*Mycobacterium tuberculosis* Infections” and “QuantiFERON-TB-Plus”. All search keywords used are listed in Additional file 1: Table S2.

### Study selection and eligibility

We included original full-text studies based on the inclusion and exclusion criteria we established, which compared, in a blinded manner, the sensitivity, specificity, or positive rate of QFT-Plus with QFT-GIT, T-SPOT.TB and TST. Studies assessing sensitivity should have included at least ten patients with active TB diagnosed by sputum culture, histopathology and biopsies (Additional file 1: Table S3). People included in studies to assess specificity should be asymptomatic and not at risk for TB infection (Additional file 1: Table S4). The population included in studies to assess positive rate must have had the following characteristics: they should be asymptomatic, with active and suspected cases of tuberculosis excluded, and they should be identified as a high-risk group (recent contacts, immunocompromised patients, with the possibility of contact and with the possibility of immunosuppression) (Additional file 1: Table S5). The definition of high-risk groups referred to the guidelines of World Health Organization (WHO) and previous study (Additional file 1: Table S6) [14, 15]. Considering the potential bias caused by the detection time, we requested that the interval of head-to-head experiments between the two experiments of the included studies be kept to 3 days.

There were eight exclusion criteria, which are detailed in the Appendix (Additional file 1: Table S7). Briefly, the following primary study types were excluded: inclusion of populations that did not meet the criteria, not head-to-head experiments, QFT-Plus was not used, and QFT-Plus was not compared with QFT-GIT, T-SPOT.TB or TST, no full text available, and a sample size of less than 10.

Two researchers (YZ and GZZ) independently screened potential studies for inclusion in the title and abstract. Disagreements were resolved by consensus with a third researcher (WS).

### Data extraction and quality assessment

Two reviewers (YZ and GZZ) independently extracted data using a standardised form designed for this study. The extracted information included the name of the first author, year of publication, title, country of study, duration of study, population investigated, patient demographics, method of testing, number of people screened at baseline, and number of positives. Disagreements were resolved by consensus and discussion with a third reviewer (WS).

Considering that the study involved three different populations, some items in the Quality Assessment of Diagnostic Accuracy Studies 2 (QUADAS-2) tool were not suitable for this study; therefore, we modified the QUADAS-2 with some quality items to improve the assessment of the diagnostic accuracy of the study [16]. Three populations (active TB patients, populations with very low risk of TB exposure, and high-risk groups) were included in this study; therefore, we developed different quality assessment criteria for the inclusion of each population based on the modified QUADAS-2 (Additional file 1: Tables S8, S10, S12) and three quality score tables based on different quality assessment criteria (Additional file 1: Tables S9, S11, S13). High-quality studies meet at least seven of the criteria, medium-quality studies meet four to six criteria, and low-quality studies meet three or fewer criteria. Three investigators (YZ, GZZ, and WLS) independently assessed the methodological quality of one-third of the studies and a fourth investigator (NC) independently reviewed these assessments. The differences were settled by consensus.

### Statistical analyses

We calculated the sensitivity, specificity and positive rate (95% confidence intervals [CIs]) for each study and summarised the results of the forest plots. RD was used to compare the differences in sensitivity, specificity and positive rate between QFT-Plus and the other three tests.

In patients with active TB, we used the age of the participants (children were defined as those aged < 18 years), TB burden of the areas and number of participants for subgroup analysis to compare QFT-Plus with QFT-GIT and used participants for subgroup analysis to compare QFT-Plus with T-SPOT.TB. TB burden of the areas considered was determined using data from the WHO website [TB profile (shinyapps.io)] and divided as follows: 1–30 per 100,000 persons; 31–100 per 100,000 persons; 100–200 per 100,000 persons and 200-per 100,000 persons. In populations with a very low risk of TB exposure, we used TB burden of the areas and number of participants for subgroup analysis to compare QFT-Plus with QFT-GIT. In high-risk populations, we used age of the participants, TB burden of the areas, number of participants and population for subgroup analysis, and when multiple TST cut-off results (5, 10, or 15 mm) were reported in the same study, the TST-5 (cut-off 5 mm) results were retained to calculate the pooled RD value.

Sensitivity, specificity and positive rate were pooled using a general linear random-effects mixed model [17]. The *I*^2^ statistic was used to assess the heterogeneity of the included studies, with *I*^2^ > 50% indicating significant heterogeneity. We assessed publication bias using “Peters” test. All p-value were two-sided. A p-value of less than 0.05 was considered to be significant [18, 19]. The meta-analysis was conducted using the “meta” package in R statistical software version 4.1.3 [20].

## Results

### Study selection and description

**Fig. 1:**
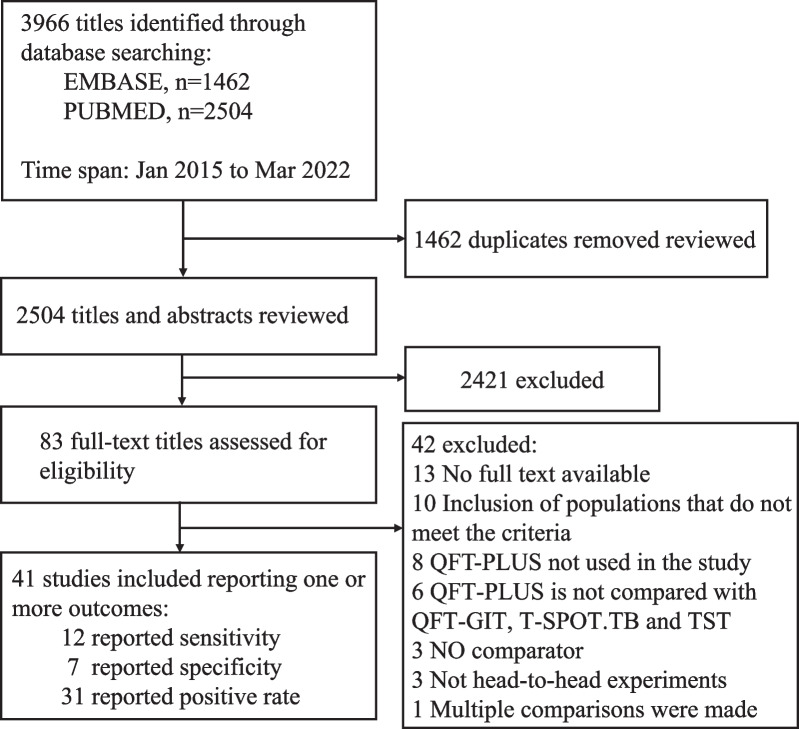
Flow diagram for search and study selection

We identified 3966 studies; 83 were selected for full-text review and 42 articles were excluded (Fig. 1 and Additional file 1: Table S14), leaving 41 studies that met our inclusion criteria. Twelve studies evaluated sensitivity [10, 21–31], seven evaluated specificity [21, 23, 24, 28, 31–33] and thirty-one evaluated positive rate [10, 22, 27, 30, 32–58].

Twelve studies compared the sensitivity of QFT-Plus with QFT-GIT in patients with active TB (Additional file 1: Table S15). Of these, three studies compared the sensitivity of QFT-Plus with T-SPOT.TB [10, 23, 28]. The patient population that was enrolled included patients from six countries and the patient age range 0–89 years.

Seven studies compared the specificity of QFT-Plus with QFT-GIT, T-SPOT.TB and TST in populations with a very low risk of TB exposure (Additional file 1: Table S16), of which two studies compared the positive rate of QFT-Plus with QFT-GIT and QFT-Plus with T-SPOT.TB [23, 28]. The patient population that was enrolled included patients from four countries and the patient age range 2.5–75 years.

Thirty-one studies compared the positive rate of QFT-Plus with QFT-GIT, T-SPOT.TB and TST in high-risk populations (Additional file 1: Table S17), and two studies compared the positive rate of QFT-Plus with QFT-GIT and QFT-Plus with T-SPOT.TB [10, 45]. One study was included twice because it involved two populations that met the criteria for a high-risk population [10]. The patient population that was enrolled included patients from 13 countries and the patient age range 2–102 years.

### Sensitivity of QFT-PLUS compared with QFT-GIT and T-SPOT.TB

We have not retrieved the original literature comparing QFT-Plus and TST in patients with active TB. Therefore, RD values were used exclusively for reporting the sensitivity of QTF-Plus versus QTF-GIT and T-SPOT.TB.

**Fig. 2:**
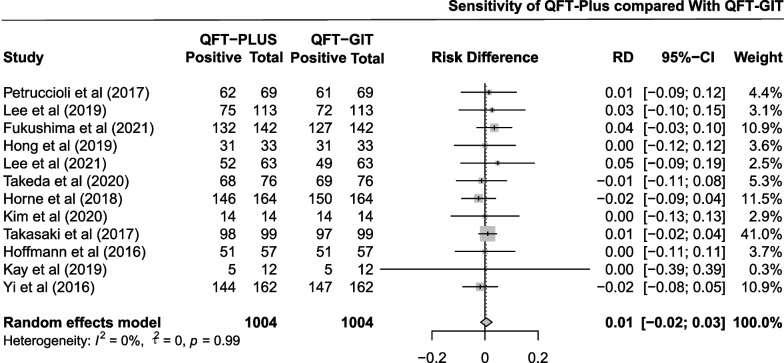
Pooled difference in sensitivity between QFT-Plus and QFT-GIT in 12 studies

The pooled difference in sensitivity between QFT-Plus and QFT-GIT was 0.01 (95% CI − 0.02 to 0.03; Fig. 2) in 12 studies with 1004 participants. As shown in Additional file 1: Fig. S1 and Table 1, the pooled estimates of sensitivity were 0.886 (95% CI 0.812 to 0.944) and 0.879 (95% CI 0.802 to 0.939) for QFT-Plus and QFT-GIT, respectively. Subgroup analysis was conducted stratified by age of the participants, TB burden of the areas, and number of participants (Additional file 1: Fig. S9).

**Table 1:** Comparison of the sensitivity, specificity and positive rate of QFT-PLUS and QFT-GIT

	Studies (n)	Participants (n)	QFT-PLUS positive	*I*^2^ (%)	QFT-GIT positive	*I*^2^ (%)	RD-test	*I*^2^ (%)
Active TB	12	1004	0.886 (0.812–0.944)	87	0.879 (0.802–0.939)	87	0.01 (− 0.02–0.03)	0
Populations with very low risk of TB exposure	5	482	0.987 (0.961–0.999)	65	0.996 (0.984–1.000)	28	0.00 (− 0.02–0.01)	0
High-risk populations	18	4617	0.235 (0.154–0.328)	97	0.228 (0.144–0.323)	97	0.02 (0.01–0.03)	0
Recent contacts	7	1391	0.311 (0.185–0.453)	97	0.286 (0.167–0.423)	97	0.02 (0.00–0.05)	0
Immunocompromised patients	4	850	0.317 (0.107–0.578)	95	0.304 (0.090–0.578)	96	0.01 (− 0.02–0.05)	0
With possibility of contact	6	2147	0.108 (0.039–0.205)	98	0.119 (0.027–0.264)	98	0.02 (0.00–0.03)	37
With possibility of immunosuppression	1	229	NA	NA	NA	NA	0.03 (− 0.05–0.12)	0

*QFT-Plus* QuantiFERON-TB Gold Plus, *QFT-GIT* QuantiFERON-TB Gold In-Tube

**Fig. 3:**
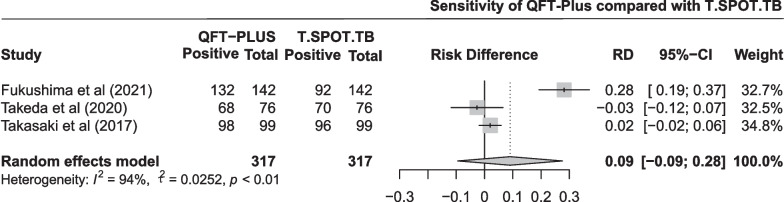
Pooled difference in sensitivity between QFT-Plus and T-SPOT.TB in 3 studies

The pooled difference in sensitivity between QFT-Plus and T-SPOT.TB was 0.09 (95% CI − 0.09 to 0.28; Fig. 3) in three studies with 317 participants. As shown in Additional file 1: Fig. S2 and Table 2, the pooled estimates of sensitivity were 0.947 (95% CI 0.873 to 0.990) and 0.872 (95% CI 0.643 to 0.991) for QFT-Plus and T-SPOT.TB, respectively. Subgroup analysis was conducted stratified by number of participants, and when the number of participants was greater than 100, QFT-Plus had a significant advantage over T-SPOT.TB (Additional file 1: Fig. S10).

**Table 2:** Comparison of the sensitivity, specificity and positive rate of QFT-PLUS and T-SPOT.TB

	Studies (n)	Participants (n)	QFT-PLUS positive	*I*^2^ (%)	T-SPOT.TB positive	*I*^2^ (%)	RD-test	*I*^2^ (%)
Active TB	3	317	0.947 (0.873–0.990)	81	0.872 (0.643–0.991)	96	0.09 (− 0.09–0.28)	96
Populations with very low risk of TB exposure	2	224	0.995 (0.959–1.000)	76	1.000 (0.996–1.000)	0	0.00 (− 0.02–0.01)	76
High-risk populations	6	2582	0.103 (0.047–0.179)	95	0.069 (0.010–0.174)	98	0.01 (− 0.01–0.04)	54
Immunocompromised patients	2	320	NA	NA	NA	NA	0.04 (0.00–0.07)	0
With possibility of contact	2	644	NA	NA	NA	NA	− 0.01 (− 0.06–0.05)	56
With possibility of immunosuppression	2	1618	NA	NA	NA	NA	0.02 (− 0.04–0.08)	74

*QFT-Plus* QuantiFERON-TB Gold Plus, *T-SPOT.TB* T-cell spot of tuberculosis assay

### Specificity of QFT-Plus compared with QFT-GIT, T-SPOT.TB and TST

**Fig. 4:**
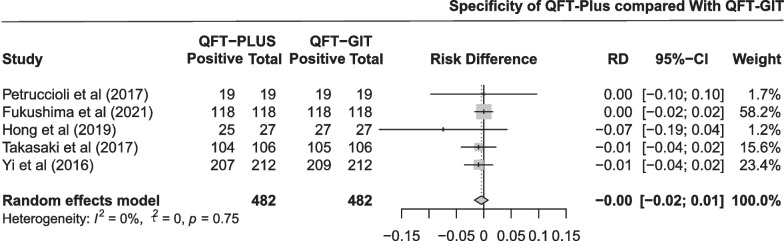
Pooled difference in specificity between QFT-Plus and QFT-GIT in 5 studies

The pooled difference in specificity between QFT-Plus and QFT-GIT was 0.00 (95% CI − 0.02 to 0.01; Fig. 4) in five studies with 482 participants. As shown in Additional file 1: Fig. S3 and Table 1, the pooled estimates of specificity were 0.987 (95% CI 0.961 to 0.999) and 0.996 (95% CI 0.984 to 1.000) for QFT-Plus and QFT-GIT, respectively. Subgroup analysis was conducted stratified by TB burden of the areas and number of participants (Additional file 1: Fig. S11).

**Fig. 5:**
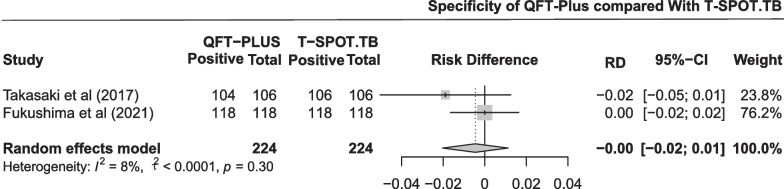
Pooled difference in specificity between QFT-Plus and T-SPOT.TB in 2 studies

The pooled difference in specificity between QFT-Plus and T-SPOT.TB was 0.00 (95% CI − 0.02 to 0.01; Fig. 5) in two studies with 224 participants. As shown in Additional file 1: Fig. S4 and Table 2, the pooled estimates of specificity were 0.995 (95% CI 0.959 to 1.000) and 1.000 (95% CI 0.996 to 1.000) for QFT-Plus and T-SPOT.TB, respectively.

**Fig. 6:**
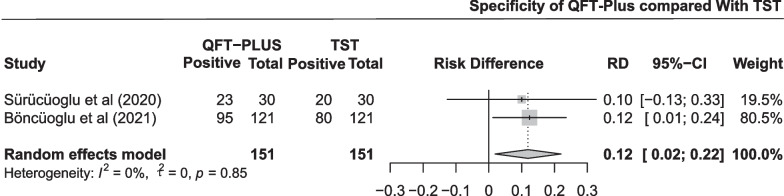
Pooled difference in specificity between QFT-Plus and TST in 2 studies

The pooled difference in specificity between QFT-Plus and TST was 0.12 (95% CI 0.02 to 0.22; Fig. 6) in two studies with 151 participants. As shown in Additional file 1: Fig. S5 and Table 3, the pooled estimates of specificity were 0.782 (95% CI 0.712 to 0.844) and 0.662 (95% CI 0.585 to 0.735) for QFT-Plus and TST, respectively.

**Table 3:** Comparison of the specificity and positive rate of QFT-PLUS and TST

	Studies (n)	Participants (n)	QFT-PLUS positive	*I*^2^ (%)	TST positive	*I*^2^ (%)	RD-test	*I*^2^ (%)
Populations with very low risk of TB exposure	2	151	0.782 (0.712–0.844)	0	0.662 (0.585–0.735)	0	0.12 (0.02–0.22)	0
High-risk populations	10	1743	0.298 (0.161–0.456)	98	0.327 (0.198–0.471)	97	− 0.03 (− 0.16–0.11)	95
Recent contacts	4	794	0.339 (0.221–0.468)	94	0.361 (0.224–0.511)	95	− 0.03 (− 0.14–0.08)	73
Immunocompromised patients	1	71	NA	NA	NA	NA	0.10 (0.00–0.20)	NA
With possibility of contact	1	158	NA	NA	NA	NA	0.01 (− 0.04–0.06)	NA
With possibility of immunosuppression	4	720	0.370 (0.083–0.722)	99	0.472 (0.327–0.620)	94	− 0.08 (− 0.43–0.27)	98

*QFT-Plus* QuantiFERON-TB Gold Plus, *TST* Tuberculin test

### Positive rate of QFT-Plus compared with QFT-GIT, T-SPOT.TB and TST

**Fig. 7:**
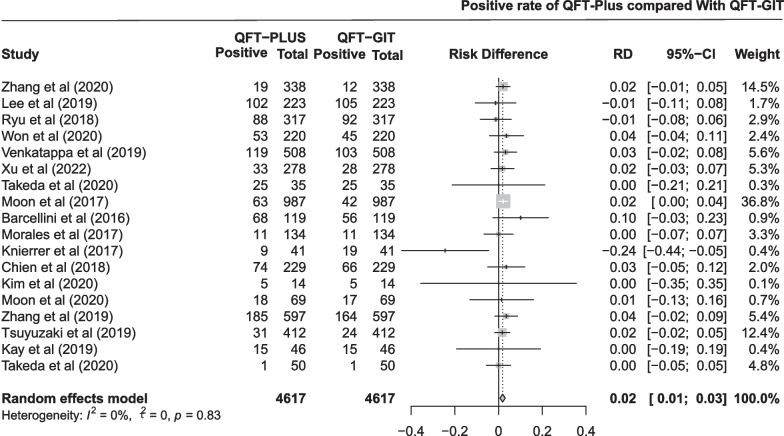
Pooled difference in positive rate between QFT-Plus and QFT-GIT in 18 studies

The pooled difference in positive rate between QFT-Plus and QFT-GIT was 0.02 (95% CI 0.01 to 0.03; Fig. 7) in 18 studies with 4617 participants. As shown in Additional file 1: Fig. S6 and Table 1, the pooled estimates of the positive rate were 0.235 (95% CI 0.154 to 0.328) and 0.228 (95% CI 0.144 to 0.323) for QFT-Plus and QFT-GIT, respectively. Subgroup analysis was conducted stratified by age of the participants, TB burden of the areas, number of participants, and population (Additional file 1: Fig. S12).

**Fig. 8:**
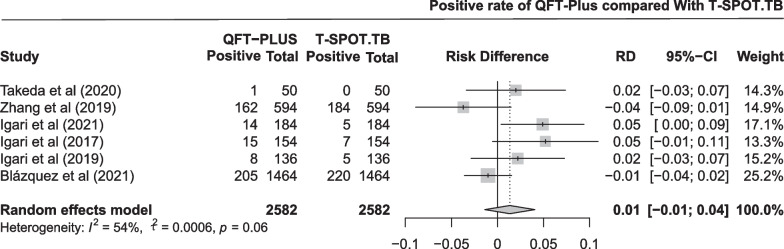
Pooled difference in positive rate between QFT-Plus and T-SPOT.TB in 6 studies

The pooled difference in the positive rate between QFT-Plus and T-SPOT.TB was 0.01 (95% CI − 0.01 to 0.04; Fig. 8) in six studies with 2582 participants. As shown in Additional file 1: Fig. S7 and Table 2, the pooled estimates of positive rate were 0.103 (95% CI 0.047 to 0.179) and 0.069 (95% CI 0.010 to 0.174) for QFT-Plus and T-SPOT.TB, respectively. Subgroup analysis was conducted stratified by age of the participants, TB burden of the areas, number of participants, and population (Additional file 1: Fig. S13).

**Fig. 9:**
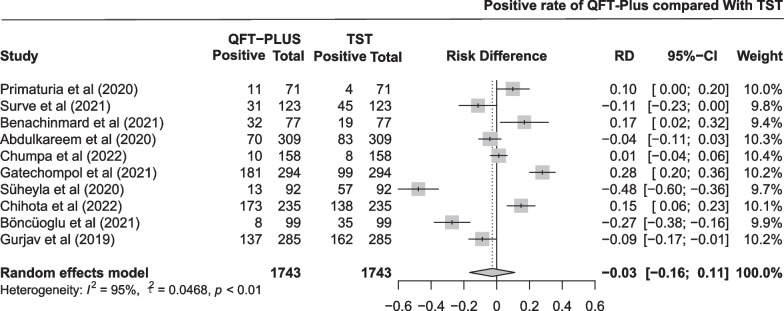
Pooled difference in positive rate between QFT-Plus and TST in 10 studies

The pooled difference in positive rate between QFT-Plus and TST was − 0.03 (95% CI − 0.16 to 0.11; Fig. 9) in 10 studies with 1743 participants. As shown in Additional file 1: Fig. S8 and Table 3, the pooled estimates of the positive rate were 0.298 (95% CI 0.161 to 0.456) and 0.327 (95% CI 0.198 to 0.471) for QFT-Plus and TST, respectively. Subgroup analysis was conducted stratified by age of the participants, TB burden of the areas, number of participants, and population (Additional file 1: Fig. S14).

The results of the sensitivity analysis showed that the results were stable (Additional file 1: Fig. S15–S17). The “Peters” test was set as a parameter for publication bias detection, enabling the following comparisons: QFT-PLUS versus QFT-GIT (p = 0.91) in patients with active TB; QFT-PLUS versus QFT-GIT (p = 0.19), and QFT-PLUS versus TST (p = 0.25) in high-risk populations. As a result, no evidence of publication bias was found (for details see Additional file 1: Table S18 and Figs. S18, S19).

## Discussion

We compared the diagnostic sensitivity, specificity and positive rate of QFT-Plus with those of QFT-GIT, T-SPOT.TB, and TST in different populations. Among the sensitivities tested in patients with active TB, no difference between QFT-Plus and GFT-GIT was identified. The sensitivity of QFT-PLUS is higher than that of T-SPOT.TB, but the 95% CI was imprecise (included zero). Among the specificity tested in populations with a very low risk of TB exposure, QFT-Plus showed hardly any difference in specificity compared to QFT-GIT and T-SPOT.TB. However, compared to TST, QFT-Plus shows significant advantages, and we speculate that it is possible that prior BCG vaccination or non-*Mycobacterium tuberculosis*-infected populations are causing many false positives in the TST test [59–61]. Among the positive rate tested in high-risk groups, the pooled positive rate was statistically significant for QFT-Plus compared to QFT-GIT, but not for T-SPOT.TB or TST.

We did not find evidence that QFT-Plus has better sensitivity and specificity than QFT-GIT. In a previous analysis, it was shown that QFT-Plus has a higher sensitivity than QFT-GIT [62]. In contrast to our results, we speculate that this may be because the inclusion populations differed, and our inclusion populations were head-to-head experimental populations that received both tests. A meta-analysis also compared the performance of QFT-Plus with QFT-GIT, and the same populations were included, they produced similar results to ours [13].

In the study, the authors asked whether QFT-Plus use might be more advantageous than QFT-GIT in an immunosuppressed population because QFT-Plus has two TB antigen tubes that stimulate IFN-γ production by CD8+ T cells and CD4+ T cells. Therefore, we conducted a subgroup analysis of high-risk populations. Results of this analysis showed that both tests performed similarly in the immunosuppressed population, with no significant advantage of QFT-PLUS use revealed. However, we can see that QFT-Plus, with its two TB antigen tubes, may have a genuine advantage in detecting positive rate in high-risk groups. It is possible that the small amount of data we included may have biased the results obtained; therefore, it is recommended that subsequent researchers continue to focus on this issue and include more data to obtain more reliable results.

There are also some limitations to our study. First, the sensitivity and specificity of our findings are underestimated because TB exposure may still be present in a population with very low risk of TB exposure and patients with active TB have partially compromised basic immunity, resulting in a reduced power to detect TB. Second, the absence of HIV infection in high-risk populations and the rare inclusion of children may have influenced our assessment of the positivity rate in high-risk populations.

The use of QFT-Plus in clinical situations can be convenient and affordable. However, with respect to convenience, the procedures for both tests are similar. With respect to affordability, the cost difference between using the QFT-Plus and QFT-GIT tests is not significant. However, in our study, we found a slight advantage of QFT-Plus over QFT-GIT in positive rate, which may not be sufficient to use QFT-Plus as a recommended method for detecting positivity rate in high-risk populations; therefore, more data need to be included in subsequent studies.

## Conclusions

The detection performance of QFT-Plus is not significantly improved compared with QFT-GIT and T-SPOT.TB, and the findings of this systematic review should encourage people to choose methods that are more convenient and economical for TB testing.

## Supplementary Information

**Additional file 1: Table S1.** PRISMA checklist. **Table S2.** Search strategy. **Table S2.** Search strategy. **Table S3.** Inclusion and partial exclusion for patients with active TB. **Table S4.** Inclusion and partial exclusion for populations with very low risk of TB exposure. **Table S5.** Inclusion and partial exclusion for high-risk populations. **Table S6.** Populations considered high-risk. **Table S7.** Details of excluded criteria. **Table S8.** QUADAS-2 adapted quality assessment criteria for patients with active TB. **Table S9.** Quality score of 12 studies for patients with active TB. **Table S10.** QUADAS-2 adapted quality assessment criteria for populations with very low risk of TB exposure. **Table S11.** Quality score of 7 studies for populations with very low risk of TB exposure. **Table S12.** QUADAS-2 adapted quality assessment criteria for high-risk groups. **Table S13.** Quality score of 31 studies for high-risk groups. **Table S14.** Reasons for exclusion of 42 studies that were read in full-text review. **Table S15.** Characteristics of the 12 studies included in the sensitivity analysis. **Table S16.** Characteristics of the 7 studies included in the specificity analysis. **Table S17.** Characteristics of the 31 studies included in the positive rates. **Table S18.** Linearregression test of funnel plot asymmetry results of QFT-PLUS compared to QFT-GIT, T-SPOT.TB and TST in three populations. **Figure S1.** Forest plot of studies estimating the sensitivity of QFT-Plus (A) and QFT-GIT (B) in patients with active tuberculosis. **Figure S2.** Forest plot of studies estimating the sensitivity of QFT-Plus (A) and T-SPOT.TB (B) in patients with active tuberculosis. **Figure S3.** Forest plot of studies estimating the specificity of QFT-Plus (A) and QFT-GIT (B) in populations with very low risk of TB exposure. **Figure S4.** Forest plot of studies estimating the specificity of QFT-Plus (A) and T-SPOT.TB (B) in populations with very low risk of TB exposure. **Figure S5.** Forest plot of studies estimating the specificity of QFT-Plus (A) and TST (B) in populations with very low risk of TB exposure. **Figure S6.** Forest plot of studies estimating the positive rate Plus (A) and QFT-GIT (B) in high-risk populations. **Figure S7.** Forest plot of studies estimating the positive rate of QFT-Plus (A) and T-SPOT.TB (B) in high-risk populations. **Figure S8.** Forest plot of studies estimating the positive rate of QFT-Plus (A) and TST (B) in high-risk populations. **Figure S9.** Forest plot of studies estimating the sensitivity in patients with active tuberculosis for age of the participants (A), TB burden of the areas (B) and number of participants (C) subgroup analysis of QFT-PLUS compared with QFT-GIT. **Figure S10.** Forest plot of studies estimating the sensitivity in patients with active tuberculosis for number of participants subgroup analysis of QFT-PLUS compared with T-SPOT.TB. **Figure S11.** Forest plot of studies estimating the Specificity in populations with very low risk of TB exposure for TB burden of the areas (A) and number of participants (B) subgroup analysis of QFT-PLUS compared with QFT-GIT. **Figure S12.** Forest plot of studies estimating the positive rate in high-risk populations for age of the participants (A), TB burden of the areas (B), number of participants (C) and population (D)subgroup analysis of QFT-PLUS compared with QFT-GIT. **Figure S13.** Forest plot of studies estimating the positive rate in high-risk populations for age of the participants (A), TB burden of the areas (B), number of participants (C) and population (D)subgroup analysis of QFT-PLUS compared with T-SPOT.TB. **Figure S14.** Forest plot of studies estimating the positive rate in high-risk populations for age of the participants (A), TB burden of the areas (B), number of participants (C) and population (D)subgroup analysis of QFT-PLUS compared with TST. **Figure S15.** Sensitivity analysis of QFT-PLUS compared to QFT-GIT (A) and T-SPOT.TB (B) in patients with active TB. **Figure S16.** Sensitivity analysis of QFT-PLUS compared to QFT-GIT (A), T-SPOT.TB (B) and TST(C) in populations with very low risk of TB exposure. **Figure S17.** Sensitivity analysis of QFT-PLUS compared to QFT-GIT (A), T-SPOT.TB (B) and TST(C) in high-risk populations. **Figure S18.** Funnel plot of QFT-PLUS compared to QFT-GIT in patients with active TB. **Figure S19.** Funnel plot of QFT-PLUS compared to QFT-GIT (A) and TST (B) in high-risk populations.

## Data Availability

The datasets used and/or analysed during the current study are available from the corresponding author on reasonable request.

## Abbreviations

QFT-GIT: QuantiFERON-TB Gold In-Tube
QFT-Plus: QuantiFERON-TB Gold Plus
T-SPOT.TB: T-cell spot of tuberculosis assay
TST: Tuberculin test
RD: Risk difference
NTM: Non-tuberculous mycobacterium
QUADAS-2: Quality Assessment of Diagnostic Accuracy Studies 2
