# Supplementary material for: Comparing the diagnostic performance of QuantiFERON-TB Gold Plus with QFT-GIT, T-SPOT.TB and TST: a systematic review and meta-analysis
Source: BMC Infect Dis. 2023 Jan 20;23:40. doi: 10.1186/s12879-023-08008-2 (PMC9862551; doi:10.1186/s12879-023-08008-2)

Additional file

Table S1：PRISMA checklist........ 2
Table S2：Search stategy 5
Table S3：Inclusion and partial exclusion for patients with active TB 7
Table S4：Inclusion and partial exclusion for populations with very low risk of TB exposure........ 7
Table S5：Inclusion and partial exclusion for high-risk populations 8
Table S6：Populations considered high-risk........ 8
Table S7：Details of excluded criteria........ 9
Table S8：QUADAS-2 adapted quality assessment criteria for patients with active TB 10
Table S9：Quality score of 12 studies for patients with active TB 11
Table S10：QUADAS-2 adapted quality assessment criteria for populations with very low risk of TB exposure........ 12
Table S11：Quality score of 7 studies for populations with very low risk of TB exposure........ 13
Table S12：QUADAS-2 adapted quality assessment criteria for high-risk groups........ 14
Table S13：Quality score of 31 studies for high-risk groups........ 15
Table S14：Reasons for exclusion of 42 studies that were read in full-text review........ 16
Table S15：Characteristics of the 12 studies included in the sensitivity analysis........ 21
Table S16：Characteristics of the 7 studies included in the specificity analysis 22
Table S17：Characteristics of the 31 studies included in the positive rates 23
Table S18：Linearregression test of funnel plot asymmetry results of QFT-PLUS compared to QFT-GIT, T-SPOT.TB and TST in three populations. 25

Figure S1：Forest plot of studies estimating the sensitivity of QFT-Plus (A) and QFT-GIT (B) in patients with active tuberculosis........ 26
Figure S2：Forest plot of studies estimating the sensitivity of QFT-Plus (A) and T-SPOT.TB (B) in patients with active tuberculosis........ 27
Figure S3：Forest plot of studies estimating the specificity of QFT-Plus (A) and QFT-GIT (B) in populations with very low risk of TB exposure........ 28
Figure S4：Forest plot of studies estimating the specificity of QFT-Plus (A) and T-SPOT.TB (B) in populations with very low risk of TB exposure........ 29
Figure S5：Forest plot of studies estimating the specificity of QFT-Plus (A) and TST (B) in populations with very low risk of TB exposure. 30
Figure S6：Forest plot of studies estimating the positive rate Plus (A) and QFT-GIT (B) in high-risk populations. 31
Figure S7：Forest plot of studies estimating the positive rate of QFT-Plus (A) and T-SPOT.TB (B) in high-risk populations........ 32
Figure S8：Forest plot of studies estimating the positive rate of QFT-Plus (A) and TST (B) in high-risk populations........ 33
Figure S9：Forest plot of studies estimating the sensitivity in patients with active tuberculosis for age of the participants (A), TB burden of the areas (B) and number of participants (C) subgroup analysis of QFT-PLUS compared with QFT-GIT. 34
Figure S10：Forest plot of studies estimating the sensitivity in patients with active tuberculosis for number of participants subgroup analysis of QFT-PLUS compared with T-SPOT.TB. 36
Figure S11：Forest plot of studies estimating the Specificity in populations with very low risk of TB exposure for TB burden of the areas (A) and number of participants (B) subgroup analysis of QFT-PLUS compared with QFT-GIT........ 37
Figure S12：Forest plot of studies estimating the positive rate in high-risk populations for age of the participants (A), TB burden of the areas (B), number of participants (C) and population (D)subgroup analysis of QFT-PLUS compared with QFT-GIT. 38
Figure S13：Forest plot of studies estimating the positive rate in high-risk populations for age of the participants (A), TB burden of the areas (B), number of participants (C) and population (D)subgroup analysis of QFT-PLUS compared with T-SPOT.TB. 42
Figure S14：Forest plot of studies estimating the positive rate in high-risk populations for age of the participants (A), TB burden of the areas (B), number of participants (C) and population (D)subgroup analysis of QFT-PLUS compared with TST........ 44
Figure S15：Sensitivity analysis of QFT-PLUS compared to QFT-GIT (A) and T-SPOT.TB (B) in patients with active TB........ 46
Figure S16：Sensitivity analysis of QFT-PLUS compared to QFT-GIT (A), T-SPOT.TB (B) and TST(C) in populations with very low risk of TB exposure 47
Figure S17：Sensitivity analysis of QFT-PLUS compared to QFT-GIT (A), T-SPOT.TB (B) and TST(C) in high-risk populations........ 48
Figure S18：Funnel plot of QFT-PLUS compared to QFT-GIT in patients with active TB........ 49
Figure S19：Funnel plot of QFT-PLUS compared to QFT-GIT (A) and TST (B) in high-risk populations........ 50

Table S1：PRISMA checklist

Section/topic	#	Checklist item	Reported on page #
**TITLE**			
Title	1	Identify the report as a systematic review, meta-analysis, or both.	1
**ABSTRACT**			
Structured summary	2	Provide a structured summary including, as applicable: background; objectives; data sources; study eligibility criteria, participants, and interventions; study appraisal and synthesis methods; results; limitations; conclusions and implications of key findings; systematic review registration number.	2
**INTRODUCTION**			
Rationale	3	Describe the rationale for the review in the context of what is already known.	3
Objectives	4	Provide an explicit statement of questions being addressed with reference to participants, interventions, comparisons, outcomes, and study design (PICOS).	4
**METHODS**			
Protocol and registration	5	Indicate if a review protocol exists, if and where it can be accessed (e.g., Web address), and, if available, provide registration information including registration number.	4
Eligibility criteria	6	Specify study characteristics (e.g., PICOS, length of follow-up) and report characteristics (e.g., years considered, language, publication status) used as criteria for eligibility, giving rationale.	5
Information sources	7	Describe all information sources (e.g., databases with dates of coverage, contact with study authors to identify additional studies) in the search and date last searched.	5
Search	8	Present full electronic search strategy for at least one database, including any limits used, such that it could be repeated.	5, Table S2
Study selection	9	State the process for selecting studies (i.e., screening, eligibility, included in systematic review, and, if applicable, included in the meta-analysis).	6
Data collection process	10	Describe method of data extraction from reports (e.g., piloted forms, independently, in duplicate) and any processes for obtaining and confirming data from investigators.	6
Data items	11	List and define all variables for which data were sought (e.g., PICOS, funding sources) and any assumptions and simplifications made.	6
Risk of bias in individual studies	12	Describe methods used for assessing risk of bias of individual studies (including specification of whether this was done at the study or outcome level), and how this information is to be used in any data	7

		synthesis.	
Summary measures	13	State the principal summary measures (e.g., risk ratio, difference in means).	7
Synthesis of results	14	Describe the methods of handling data and combining results of studies, if done, including measures of consistency (e.g., I^2) for each meta-analysis.	7
Risk of bias across studies	15	Specify any assessment of risk of bias that may affect the cumulative evidence (e.g., publication bias, selective reporting within studies).	8
Additional analyses	16	Describe methods of additional analyses (e.g., sensitivity or subgroup analyses, meta-regression), if done, indicating which were pre-specified.	8
**RESULTS**			
Study selection	17	Give numbers of studies screened, assessed for eligibility, and included in the review, with reasons for exclusions at each stage, ideally with a flow diagram.	8
Study characteristics	18	For each study, present characteristics for which data were extracted (e.g., study size, PICOS, follow-up period) and provide the citations.	8
Risk of bias within studies	19	Present data on risk of bias of each study and, if available, any outcome level assessment	Figure S15-S17
Results of individual studies	20	For all outcomes considered (benefits or harms), present, for each study: (a) simple summary data for each intervention group (b) effect estimates and confidence intervals, ideally with a forest plot.	8-11, Figure S1-S8
Synthesis of results	21	Present results of each meta-analysis done, including confidence intervals and measures of consistency.	8-11
Risk of bias across studies	22	Present results of any assessment of risk of bias across studies.	Figure S15-S19
Additional analysis	23	Give results of additional analyses, if done (e.g., sensitivity or subgroup analyses, meta-regression).	Table S18 and Figure S15-S19
**DISCUSSION**			
Summary of evidence	24	Summarize the main findings including the strength of evidence for each main outcome; consider their relevance to key groups (e.g., healthcare providers, users, and policy makers).	12-13
Limitations	25	Discuss limitations at study and outcome level (e.g., risk of bias), and at review-level (e.g., incomplete retrieval of identified research, reporting bias).	13-14
Conclusions	26	Provide a general interpretation of the results in the context of other evidence, and implications for future research.	14

FUNDING

Funding	27	Funding: Our study was financed by the Reserve Talent Development Project of Kunming Health Science and Technology (NO. 2022-SW [Reserve Talents]-006).

From: Moher D, Liberati A, Tetzlaff J, Altman DG, The PRISMA Group (2009). Preferred Reporting Items for Systematic Reviews and Meta-Analyses: The PRISMA Statement. PLoS Med 6(6): e1000097. DOI:10.1371/journal.pmed1000097

Table S2：Search stategy

Pubmed

ID	Search
#1	Mycobacterium tuberculosis Infections
#2	Mycobacterium tuberculosis Infection
#3	Kochs Disease
#4	Koch's Disease
#5	Koch Disease
#6	Tuberculosis
#7	Latent Tuberculosis
#8	TB
#9	Latent TB
#10	1 or 2 or 3 or 4 or 5 or 6 or 7 or 8 or 9
#11	Interferon-gamma Release Tests
#12	IGRA
#13	QFT-Plus
#14	QuantiFERON-TB-Plus
#15	Interferon gamma assay
#16	11 or 12 or 13 or 14 or 15
#17	10 AND 16

Embase

ID	Search
#1	Mycobacterium tuberculosis Infections
#2	Mycobacterium tuberculosis Infection
#3	Kochs Disease
#4	Koch's Disease
#5	Koch Disease
#6	Tuberculosis
#7	Latent Tuberculosis
#8	TB
#9	Latent TB
#10	1 or 2 or 3 or 4 or 5 or 6 or 7 or 8 or 9
#11	Interferon-gamma Release Tests
#12	IGRA
#13	QFT-Plus
#14	QuantiFERON-TB-Plus
#15	Interferon gamma assay
#16	11 or 12 or 13 or 14 or 15
#17	10 AND 16

Table S3：Inclusion and partial exclusion for patients with active TB

	Have no bias	Might have bias (no score at quality assessment)	Have bias (exclude)
Active TB	They were all tested by sputum culture and showed to be positive.	The population included in this study had sputum culture tests that were not positive, but had other clinical tests that were positive	1. Latent infection and normal population not fully excluded.
Head-to-head test	The aim of this study was to compare QFT-PLUS with QFT-GIT and T-SPOT.TB, each participant was tested within 3 days.	The aim of this study was to compare QFT-PLUS with QFT-GIT and T-SPOT.TB, but each participant was tested within 3-4 weeks or no time interval between the two tests was referenced.	1. The time interval between tests was more than 4 weeks.<br>2. It is not the purpose of this article to compare QFT-PLUS with QFT-GIT and T-SPOT.TB, and there is no reference to the time interval between these tests.<br>3. Only QFT-PLUS positive patients were tested for QFT-GIT or T-SPOT.TB, or only QFT-GIT or T-SPOT.TB positive patients were tested for QFT-PLUS.

Table S4：Inclusion and partial exclusion for populations with very low risk of TB exposure

	Have no bias	Might have bias (no score at quality assessment)	Have bias (exclude)
Populations with very low risk of TB exposure	They should be asymptomatic and not at risk of infection	They are at slight risk of infection	1. Active and/or suspected cases are not excluded.<br>2. There is a definite risk of infection that could lead to active and/or suspected cases of TB
Head-to-head test	The aim of this study was to compare QFT-PLUS with QFT-GIT, T-SPOT.TB and TST, each participant was tested within 3 days.	The aim of this study was to compare QFT-PLUS with QFT-GIT, T-SPOT.TB and TST, but each participant was tested within 3-4 weeks or no time interval between the two tests was referenced.	1. The time interval between tests was more than 4 weeks.<br>2. It is not the purpose of this article to compare QFT-PLUS with QFT-GIT, T-SPOT.TB and TST, and there is no reference to the time interval between these tests.<br>3. Only QFT-PLUS positive patients were tested for QFT-GIT or T-SPOT.TB or TST, or only QFT-GIT or T-SPOT.TB or TST positive patients were tested for QFT-PLUS.

Table S5：Inclusion and partial exclusion for high-risk populations

	Have no bias	Might have bias (no score at quality assessment)	Have bias (exclude)
high-risk populations	They should be asymptomatic and indicate that active and/or suspected cases of tuberculosis have been excluded.	The aim of this study was to screen for LTBI, but there was no reference to whether active and/or suspected cases of TB were excluded.	1. Active and/or suspected cases are not excluded.<br>2. The purpose of this article is not to screen for LTBI and there is no reference to whether active tuberculosis and/or suspected cases have been excluded<br>3. Only QFT-PLUS positive, QFT-PLUS negative, QFT-GIT, T-SPOT.TB and TST positive, or QFT-GIT, T-SPOT.TB and TST negative people were included
Head-to-head test	The aim of this study was to compare QFT-PLUS with QFT-GIT, T-SPOT.TB and TST, each participant was tested within 3 days.	The aim of this study was to compare QFT-PLUS with QFT-GIT, T-SPOT.TB and TST, but each participant was tested within 3-4 weeks or no time interval between the two tests was referenced.	1. The time interval between tests was more than 4 weeks.<br>2. It is not the purpose of this article to compare QFT-PLUS with QFT-GIT, T-SPOT.TB and TST, and there is no reference to the time interval between these tests.<br>3. Only QFT-PLUS positive patients were tested for QFT-GIT or T-SPOT.TB or TST, or only QFT-GIT or T-SPOT.TB or TST positive patients were tested for QFT-PLUS.

Table S6：Populations considered high-risk

Recent contacts	Both close/household and casual
Immunocompromised patients	1.People living with HIV; 2. Chronic renal failure and/or hemodialysis; 3. Transplant recipients (organ or hematopoietic stem cell); 4. Drug and/or alcohol abusers; 5. Cancer (all types); 6. Malnourished (BMI ≤ 18.5 kg/m); 7. Silicosis.
With the possibility of contact	1.Occupational risk (e.g. healthcare worker); 2. Immigrants or refugees; 3. Army personnel.
With the possibility of immunosuppression	1.IMID; 2. Prisoners; 3. Children; 4. Nursing home residents; 5. Homeless.

Table S7： Details of excluded criteria

Patients	1.We excluded studies that inclusion of populations do not meet the criteria.<br>2.We excluded studies that not head-to-head experiments.
Test conduct	1.We excluded studies that QFT-PLUS was not used.<br>2.We excluded studies that QFT-PLUS was not compared with QFT-GIT, T-SPOT.TB and TST<br>3.We excluded the studies that no comparator.<br>4. We excluded the studies that multiple comparisons were made.
Flow and outcome	1.We excluded the studies that no full text available.<br>2.We excluded studies with a sample size of less than 10.

Table S8：QUADAS-2 adapted quality assessment criteria for patients with active TB

Patient select bias	Q1: Was a consecutive or random sample of patients enrolled?<br>We scored “Yes” if a consecutive or random sample of eligible patients was Enrolled; “No” if patients were selected by convenience; and “Unclear” if the study did not report the manner in which patients were enrolled.<br>Q2: Were all patients included have gold standard testing?<br>We scored “Yes” if all patients received the gold standard; “No” if there are patients does not receive the gold standard and other clinical tests prove negative or no clinical tests prove; and “Unclear” if there are patients who did not receive the gold standard, but other clinical tests proved positive.<br>Q3: Did the study have appropriate exclusions?<br>We scored “Yes” if current active TB and people with TB symptom were excluded, or they were grouped separately, or the definition of LTBI was stated as asymptomatic; “No” if it was unclear that active TB and people with TB symptom were excluded; and “Unclear” if only active TB or people with TB symptom were excluded or grouped.
Test conduct bias	Q4: Was the study conducted to compare QFT-PLUS with QFT-GIT, T.SPOT.TB and TST tests??<br>We scored “Yes” if the study was conducted to compare QFT-PLUS with QFT-GIT, T.SPOT.TB and TST tests?, “No” if the study was not conduced to compare QFT-PLUS with QFT-GIT, T.SPOT.TB and TST tests?; “Unclear” if this was not stated or stated inadequately.<br>Q5: If a threshold is used, was it confirmed beforehand?<br>We scored “Yes” if the threshold values are used and predetermined, “No” if no prior determination; “Unclear” if there is no description.<br>Q6: Was how the tests were conducted and interpreted adequately described?<br>We scored “Yes” if the tests were conducted and interpreted adequately described, such as the cut-off, time interval of the results were read, and manufacturer information, or “performed as the manufacturer’s guidelines” was stated; and “No” if those information were not stated or stated inadequately.<br>Q7: Was there an appropriate interval between QFT-PLUS with QFT-GIT, T.SPOT.TB and TST tests?<br>We scored “Yes” if the two tests were paired or performed within 3 days, or "paired" comparison, “head-to-head” comparison was reported, and “No” if the interval was more than 3 days and less than 4 weeks; “Unclear” if this was not stated.
Flow and outcome	Q8: Were the results of two tests interpreted without knowledge of each other?<br>We scored “Yes” if the results of two tests were interpreted without knowledge of each other, or one test was interpreted blinded to another; “No” if blinding to test results were not done; and “Unclear” if this was not stated.<br>Q9: Were all patients included in the analysis?<br>We answered this question by comparing the number of participants included in the study and the number of individuals included in the 2x2 tables, test agreement data, or flow diagram. We scored ‘Yes’ if the number of participants enrolled was stated and corresponded to the number included in the analysis or if exclusions were adequately described. We scored “No” if there were participants missing or excluded from the analysis and there was no explanation given. We scored “Unclear” if we could not tell, e.g. because the number of participants enrolled and/or number of participants included in the analysis was not clearly stated.

Table S9：Quality score of 12 studies for patients with active TB

**Study**	**1**	**2**	**3**	**4**	**5**	**6**	**7**	**8**	**9**	**Quality score**
Petruccioli et al 2017	0	0	0	1	1	1	0	1	1	5
Lee et al 2019	0	0	1	1	1	1	1	0	1	6
Fukushima et al 2021	0	0	1	1	1	1	1	0	1	6
Hong et al 2019	0	0	1	1	1	1	0	0	1	5
Lee et al 2021	0	0	1	1	1	0	1	0	1	5
Takeda et al 2020	0	1	1	1	1	1	1	0	1	7
Horne et al 2018	1	1	0	1	1	1	1	0	1	7
Kim et al 2020	0	1	0	1	1	1	1	0	1	6
Takasaki et al 2017	0	0	1	1	1	1	1	1	0	6
Hoffmann et al 2016	0	0	1	0	1	1	0	0	1	4
Kay et al 2019	0	0	1	0	1	0	0	0	1	3
Yi et al 2016	0	1	1	1	1	1	1	1	1	8

Table S10：QUADAS-2 adapted quality assessment criteria for populations with very low risk of TB exposure

Patient select bias	Q1: Is the population included in this study at little or no risk of infection with tuberculosis?<br>We scored “Yes” if they live and work in an environment where they are not exposed to TB; “No” if they have exposure to TB; and “Unclear” if the study did not report.<br>Q2: Was a consecutive or random sample of patients enrolled?<br>We scored “Yes” if a consecutive or random sample of eligible patients was Enrolled; “No” if patients were selected by convenience; and “Unclear” if the study did not report the manner in which patients were enrolled.<br>Q3: Did the study have appropriate exclusions?<br>We scored “Yes” if current active TB and people with TB symptom were excluded, or they were grouped separately, or the definition of LTBI was stated as asymptomatic; “No” if it was unclear that active TB and people with TB symptom were excluded; and “Unclear” if only active TB or people with TB symptom were excluded or grouped.
Test conduct bias	Q4: Was the study conducted to compare QFT-PLUS with QFT-GIT, T.SPOT.TB and TST tests??<br>We scored “Yes” if the study was conducted to compare QFT-PLUS with QFT-GIT, T.SPOT.TB and TST tests?, “No” if the study was not conduced to compare QFT-PLUS with QFT-GIT, T.SPOT.TB and TST tests?; “Unclear” if this was not stated or stated inadequately.<br>Q5: Was how the tests were conducted and interpreted adequately described?<br>We scored “Yes” if the tests were conducted and interpreted adequately described, such as the cut-off, time interval of the results were read, and manufacturer information, or “performed as the manufacturer’s guidelines” was stated; and “No” if those information were not stated or stated inadequately.<br>Q6: Was there an appropriate interval between QFT-PLUS with QFT-GIT, T.SPOT.TB and TST tests?<br>We scored “Yes” if the two tests were paired or performed within 3 days, or "paired" comparison, “head-to-head” comparison was reported, and “No” if the interval was more than 3 days and less than 4 weeks; “Unclear” if this was not stated.<br>Q7: If a threshold is used, was it confirmed beforehand?<br>We scored “Yes” if the threshold values are used and predetermined, “No” if no prior determination; “Unclear” if there is no description.
Flow and outcome	Q8: Were the results of two tests interpreted without knowledge of each other?<br>We scored “Yes” if the results of two tests were interpreted without knowledge of each other, or one test was interpreted blinded to another; “No” if blinding to test results were not done; and “Unclear” if this was not stated.<br>Q9: Were all patients included in the analysis?<br>We answered this question by comparing the number of participants included in the study and the number of individuals included in the 2x2 tables, test agreement data, or flow diagram. We scored ‘Yes’ if the number of participants enrolled was stated and corresponded to the number included in the analysis or if exclusions were adequately described. We scored “No” if there were participants missing or excluded from the analysis and there was no explanation given. We scored “Unclear” if we could not tell, e.g. because the number of participants enrolled and/or number of participants included in the analysis was not clearly stated.

Table S11：Quality score of 7 studies for populations with very low risk of TB exposure

**Study**	**1**	**2**	**3**	**4**	**5**	**6**	**7**	**8**	**9**	**Quality score**
Petruccioli et al 2017	1	0	0	1	1	0	1	0	1	5
Fukushima et al 2021	1	0	0	1	1	1	1	1	1	7
Hong et al 2019	1	0	1	1	1	0	1	0	1	6
Takasaki et al 2017	1	0	1	1	1	1	1	0	1	7
Yi et al 2016	1	0	1	1	1	1	1	1	1	8
Sürücüoğlu et al 2020	0	0	0	1	1	0	1	0	1	4
Böncüoğlu et al 2021	0	0	0	1	1	0	1	0	1	4

Table S12: QUADAS-2 adapted quality assessment criteria for high-risk groups

Patient select bias	Q1: Was the study conducted to screen for LTBI?<br>We scored “Yes” if the study was conduced to screen for LTBI; and “No” if the study was not conduced to screen for LTBI; “Unclear” if this was not stated or stated inadequately.<br>Q2: Was a consecutive or random sample of patients enrolled?<br>We scored “Yes” if a consecutive or random sample of eligible patients was Enrolled; “No” if patients were selected by convenience; and “Unclear” if the study did not report the manner in which patients were enrolled.<br>Q3: Did the study have appropriate exclusions?<br>We scored “Yes” if current active TB and people with TB symptom were excluded, or they were grouped separately, or the definition of LTBI was stated as asymptomatic; “No” if it was unclear that active TB and people with TB symptom were excluded; and “Unclear” if only active TB or people with TB symptom were excluded or grouped.
Test conduct bias	Q4: Was the study conducted to compare QFT-PLUS with QFT-GIT, T.SPOT.TB and TST tests??<br>We scored “Yes” if the study was conducted to compare QFT-PLUS with QFT-GIT, T.SPOT.TB and TST tests?, “No” if the study was not conduced to compare QFT-PLUS with QFT-GIT, T.SPOT.TB and TST tests?; “Unclear” if this was not stated or stated inadequately.<br>Q5: Was how the tests were conducted and interpreted adequately described?<br>We scored “Yes” if the tests were conducted and interpreted adequately described, such as the cut-off, time interval of the results were read, and manufacturer information, or “performed as the manufacturer’s guidelines” was stated; and “No” if those information were not stated or stated inadequately.<br>Q6: Was there an appropriate interval between QFT-PLUS with QFT-GIT, T.SPOT.TB and TST tests?<br>We scored “Yes” if the two tests were paired or performed within 3 days, or "paired" comparison, “head-to-head” comparison was reported, and “No” if the interval was more than 3 days and less than 4 weeks; “Unclear” if this was not stated.<br>Q7: If a threshold is used, was it confirmed beforehand?<br>We scored “Yes” if the threshold values are used and predetermined, “No” if no prior determination; “Unclear” if there is no description.
Flow and outcome	Q8: Were the results of two tests interpreted without knowledge of each other?<br>We scored “Yes” if the results of two tests were interpreted without knowledge of each other, or one test was interpreted blinded to another; “No” if blinding to test results were not done; and “Unclear” if this was not stated.<br>Q9: Were all patients included in the analysis?<br>We answered this question by comparing the number of participants included in the study and the number of individuals included in the 2x2 tables, test agreement data, or flow diagram. We scored ‘Yes’ if the number of participants enrolled was stated and corresponded to the number included in the analysis or if exclusions were adequately described. We scored “No” if there were participants missing or excluded from the analysis and there was no explanation given. We scored “Unclear” if we could not tell, e.g. because the number of participants enrolled and/or number of participants included in the analysis was not clearly stated.

Table S13：Quality score of 31 studies for high-risk groups

Study	1	2	3	4	5	6	7	8	9	Quality score
Zhang et al 2020	1	1	1	0	1	1	1	0	1	7
Lee et al 2019	0	0	1	1	1	1	1	1	0	6
Ryu et al 2018	1	1	0	1	1	1	1	0	1	7
Won et al 2020	1	0	0	1	1	1	1	0	1	6
Venkatappa et al 2019	1	0	0	1	1	1	1	0	1	6
Xu et al 2022	0	1	1	1	1	1	1	0	1	7
Takeda et al 2020	0	0	1	1	1	1	1	0	1	6
Moon et al 2017	1	1	0	1	1	1	1	0	1	7
Barcellini et al 2016	1	0	0	1	1	1	1	0	1	6
Morales et al 2017	1	1	0	1	1	1	1	0	1	7
Knierrer et al 2017	1	0	0	1	1	1	1	0	1	6
Chien et al 2018	1	0	1	1	1	1	1	0	1	6
Kim et al 2020	1	0	0	1	1	1	1	0	1	6
Moon et al 2020	0	0	0	1	0	1	1	0	1	4
Zhang et al 2019	0	1	1	0	1	0	1	0	0	4
Tsuyuzaki et al 2019	1	0	1	1	1	1	1	0	1	7
Kay et al 2019	0	0	0	1	1	0	0	0	1	3
Takeda et al 2020	1	0	1	1	1	1	1	0	1	7
Igari et al 2021	1	0	0	1	1	1	1	0	1	6
Igari et al 2017	1	0	0	1	1	1	1	0	1	6
Igari et al 2019	1	0	0	1	1	1	1	0	1	6
Blázquez et al 2021	1	0	0	1	1	1	1	0	1	6
Primaturia et al 2020	0	1	1	1	1	0	1	0	1	6
Surve et al 2021	1	0	1	1	1	0	1	0	1	6
Benachinmard et al 2021	1	0	0	1	1	0	1	0	1	5
Abdulkareem et al 2020	1	0	1	0	1	0	1	0	1	5
Chumpa et al 2022	1	0	1	1	1	0	1	0	1	6
Gatechompol et al 2021	1	0	1	0	1	0	1	1	1	6
Süheyla et al 2020	1	0	0	1	1	0	1	0	1	5
Chihota et al 2022	0	0	0	1	1	0	1	0	1	4
Böncüoğlu et al 2021	0	0	0	1	1	0	1	0	1	4
Gurjav et al 2019	0	0	0	0	1	0	1	0	1	3

Table S14：Reasons for exclusion of 42 studies that were read in full-text review

	First author	Year	Journal	Title	Reason
1	Daisy Y.	2022	Journal of the American Academy of Dermatology	A prospective cohort study comparing the performance of interferon gamma release assays in autoimmune skin diseases	No full text available
2	Igari H.	2017	American Journal of Respiratory and Critical Care Medicine	The analysis of quantiferon-TB gold plus (4th generation QFT) in comparison with TSPOT in rheumatoid arthritis for latent tuberculosis infection	No full text available
3	Castellani C.	2021	Annals of the Rheumatic Diseases	Are interferon-gamma release assays reliable to detect tuberculosis infection in patients with rheumatoid arthritis treated with janus kinase inhibitors	No full text available
4	Ping-Huai W.	2020	Scientific reports	CD4 response of QuantiFERON-TB Gold Plus for positive consistency of latent tuberculosis infection in patients on dialysis	Multiple comparisons were made
5	Fukushima K.	2017	American Journal of Respiratory and Critical Care Medicine	Clinical comparison study of t-spot, QFT-gold and QFT-plus in the detection of active pulmonary TB in Japan	No full text available
6	Lu A.	2019	Experimental and therapeutic medicine	Clinical value of interferon-γ release assay in the diagnosis of active tuberculosis	Inclusion of populations that do not meet the criteria
7	Daniel E.A.	2020	BMC Infectious Diseases	Comparative analysis of the performance of Quantiferon-TB Gold Plus and Quantiferon-TB Gold In-Tube Assays in a high TB prevalence setting	No full text available
8	Sofia S.	2021	Pulmonology	Comparing the cost-effectiveness of two screening strategies for latent tuberculosis infection in Portugal	No comparator
9	Alok kumar M.	2021	Tuberculosis research and treatment	Comparison of Interferon-Gamma Release Assay and Tuberculin Skin Test for the Screening of Latent Tuberculosis in Inflammatory Bowel Disease Patients: Indian Scenario	QFT-Plus not used in this study
10	Keita T.	2020	Journal of infection and chemotherapy	Comparison of QuantiFERON-TB Gold Plus and T-SPOT.TB in the Diagnosis of Active	No full text available

				Tuberculosis	
11	Ock-Hwa K.	2020	PloS one	Comparison of the change in QuantiFERON-TB gold plus and QuantiFERON-TB gold in-tube results after preventive therapy for latent tuberculosis infection	Inclusion of populations that do not meet the criteria
12	Elitza S T.	2018	Journal of clinical microbiology	Comparison of the QuantiFERON-TB Gold Plus and QuantiFERON-TB Gold In-Tube Interferon Gamma Release Assays in Patients at Risk for Tuberculosis and in Health Care Workers	Inclusion of populations that do not meet the criteria
13	Laura H.	2022	Diagnostic microbiology and infectious disease	Comparison of the QuantiFERON-TB® Gold Plus on LIAISON® XL and T-SPOT.TB for the diagnosis of latent Mycobacterium tuberculosis infection in a low tuberculosis incidence population	Inclusion of populations that do not meet the criteria
14	Portell Rigo I.M.	2021	Clinical Chemistry and Laboratory Medicine	Concordance between interferon-gamma release assay (IGRA) and tuberculin skin test in the diagnosis of latent tuberculosis among immigrants	No full text available
15	Roxnan Mansour G.	2021	BMC pediatrics	Diagnosis of latent tuberculosis infection among pediatric household contacts of Iranian tuberculosis cases using tuberculin skin test, IFN- γ release assay and IFN-γ-induced protein-10	Inclusion of populations that do not meet the criteria
16	Martinez-Lopez D.	2021	Arthritis and Rheumatology	Epidemiology of latent tuberculosis infection in patients with rheumatic immunemediated diseases. Single university study of 1117 patients	No full text available
17	Hoon Hee L.	2021	Clinical Rheumatology	Evaluation of a lateral flow assay-based IFN-γ release assay as a point-of-care test for the diagnosis of latent tuberculosis infection	QFT-PLUS is not compared with QFT-GIT, T-SPOT.TB and TST
18	F Stieber.	2021	The International Journal of Tuberculosis and Lung Disease	Evaluation of a lateral-flow nanoparticle fluorescence assay for TB infection diagnosis	QFT-PLUS is not compared with QFT-GIT, T-SPOT.TB and TST
19	K Fukushima.	2021	Pulmonology	First clinical evaluation of the QIAreachTM QuantiFERON-	QFT-PLUS is not

				TB for tuberculosis infection and active pulmonary disease	compared with QFT-GIT, T-SPOT.TB and TST
20	Shafeque A.	2020	Journal Clinical Microbiology	Fourth-generation quanti FERON-TB gold plus: What is the evidence?	QFT-Plus not used in this study, No comparator
21	Suu Soo A.	2021	Journal of clinical medicine	Frequency and factors of indeterminate quantiferon-tb gold in-tube and quantiferon-tb gold plus test results in rheumatic diseases	Not head-to-head experiments
22	Kyuwon K.	2021	Frontiers in medicine	Frequency of Positive Conversion of Interferon-Gamma Release Assay Results Among Patients With Inflammatory Bowel Disease Treated With Non-tumor Necrosis Factor Inhibitors	No comparator
23	R M Ghanaiee	2022	New microbes and new infections	Household contact investigation for the detection of active tuberculosis and latent tuberculosis: A comprehensive evaluation in two high-burden provinces in Iran	Inclusion of populations that do not meet the criteria
24	Bagheri Z.	2021	Molecular Genetics Microbiology and Virology	Inconsistency of QuantiFERON-TB Gold Test and Tuberculin Skin Test Results in the Evaluation of Latent Tuberculosis Infection in Health Care Workers	QFT-Plus not used in this study
25	Julia M S.	2021	International Journal of Infectious Diseases	Interferon gamma release assays for detection of latent Mycobacterium tuberculosis in older Hispanic people	Inclusion of populations that do not meet the criteria
26	Jaewan J.	2021	Journal of Clinical Medicine	Is the new interferon-gamma releasing assay beneficial for the diagnosis of latent and active mycobacterium tuberculosis infections in tertiary care setting?	QFT-PLUS is not compared with QFT-GIT, T-SPOT.TB and TST
27	Chiara Della B.	2020	International Journal of Infectious Diseases	LIOFeron®TB/LTBI: A novel and reliable test for LTBI and tuberculosis	QFT-PLUS is not compared with QFT-GIT, T-SPOT.TB and TST
28	Elisa P.	2016	International Journal of Mycobacteriology	Modulation of interferon-gamma response to QuantiFERON-TB-plus	No full text available

				detected by enzyme-linked immunosorbent assay in patients with active and latent tuberculosis infection	
29	E D Pieterman.	2018	Tuberculosis (Edinburgh, Scotland)	A multicentre verification study of the QuantiFERON®-TB Gold Plus assay	Inclusion of populations that do not meet the criteria
30	Christine A.	2021	The Pediatric Infectious Disease Journal	Non-IFNγ Whole Blood Cytokine Responses to Mycobacterium tuberculosis Antigens in HIV-exposed Infants	Not head-to-head experiments
31	Songsri K.	2015	Journal of Clinical and Diagnostic Research	Performance of a rapid strip test for the serologic diagnosis of latent tuberculosis in children	QFT-Plus not used in this study
32	Shu C.	2019	American Journal of Respiratory and Critical Care Medicine	The positive status of latent tuberculosis infection is more consistent by quantiFERON-TB gold plus than that by quantiFERON-TB gold In-tube	No full text available
33	Carrizales-Luna J.P.	2019	Annals of the Rheumatic Diseases	Quantiferon gold-plus and tuberculin skin test reactivity predictors in patients with rheumatoid arthritis	No full text available
34	Chien J.-Y.	2018	Respirology	Quantiferon-TB gold plus is more sensitive than quantiferon-TB gold in-tube for latent tuberculosis infection among residents in long-term care facility	No full text available
35	Dagmara B-T.	2021	Polish Journal of Microbiology	QuantiFERON-TB Gold Plus Test in Diagnostics of Latent Tuberculosis Infection in Children Aged 1-14 in a Country with a Low Tuberculosis Incidence	Not head-to-head experiments
36	Ciaxia B.	2021	Microbiology Spectrum	Retrospective Performance Analyses of over Two Million U.S. QuantiFERON Blood Sample Results	Inclusion of populations that do not meet the criteria
37	S A R Siegel	2018	Journal of Clinical Microbiology	QuantiFERON-TB Plus, a Neifw-Generation Interferon Gamma Release Assay	Inclusion of populations that do not meet the criteria
38	Salman S.	2020	Pathology	Spot on! evaluation of T-spot.TB following indeterminant quantiferon TB-gold plus in screening for latent tuberculosis	No full text available

39	Ramesh B.	2021	International Journal of Infectious Diseases	Stages of pregnancy and HIV affect diagnosis of tuberculosis infection and Mycobacterium tuberculosis (MTB)-induced immune response: Findings from PRACHITi, a cohort study in Pune, India: Stages of Pregnancy and HIV status influence TB diagnostic accuracy	QFT-Plus not used in this study
40	Ki Wook Y.	2016	Korean Journal of Pediatrics	Usefulness of interferon-γ release assay for the diagnosis of latent tuberculosis infection in young children	QFT-Plus not used in this study
41	Rajan S.	2021	International Journal of Rheumatic Diseases	Utility of tuberculin skin test and QuantiFERON TB gold-plus in the screening of latent tuberculosis infection in refractory spondyloarthritis patients	QFT-Plus not used in this study
42	Oh Joo K.	2022	Journal of Microbiology, Immunology, and Infection	Performance evaluation of newly developed fluorescence immunoassay-based interferon-gamma release assay for the diagnosis of latent tuberculosis infection in healthcare workers	QFT-PLUS is not compared with QFT-GIT, T-SPOT.TB and TST

Table S15：Characteristics of the 12 studies included in the sensitivity analysis

**First author, Year**	**Setting**	**TB burden of the areas**	**Comparator(s)**	**No. of participants**	**Females**	**Median age (IQR)**	**Risk of bias**
PetruccioliE, 2017	Italy	0-30	QFT-GIT	69	28(41%)	35(28-44)	medium
Meng-Rui Lee, 2019	Taiwan, China	31-100	QFT-GIT	113	49(43.4%)	NS	medium
Kiyoyasu Fukushima, 2021	Japan	0-30	QFT-GIT and T.SPOT.TB	142	60(42.3%)	84(76-89)	medium
Ji Young Hong, 2019	South Korea	31-100	QFT-GIT	33	4(12.1%)	17(17-24)	medium
Jung-Kyu Lee, 2021	South Korea	31-100	QFT-GIT	63	15(23.8%)	NS	medium
Keita Takeda, 2020	Japan	0-30	QFT-GIT and T.SPOT.TB	76	26 (34.2%)	57.7(17-96)	low
D. J. Horne, 2018	USA and Japan	0-30	QFT-GIT	164	64(39%)	NS	low
Soo Han Kim, 2020	South Korea	31-100	QFT-GIT	14	NS	NS	medium
Jin Takasaki, 2017	Japan	0-30	QFT-GIT and T.SPOT.TB	99	34(34.3%)	42 (29-55)	medium
Harald Hoffmann, 2016	Germany	0-30	QFT-GIT	57	NS	NS	medium
Alexander W. Kay, 2019	USA	0-30	QFT-GIT	12	5 (42%)	NS	high
Lina Yi, 2016	Japan	0-30	QFT-GIT	162	33(20.4%)	59 (39-70)	low

IQR, interquartile range; QFT-GIT, QuantiFERON-TB Gold In-Tube; T.SPOT.TB, T-cell spot of tuberculosis assay

Table S16：Characteristics of the 7 studies included in the specificity analysis

First author, Year	Setting	TB burden of the areas	Comparator(s)	No. of participants	Females	Median age (IQR)	Risk of bias
Petruccioli E, 2017	Italy	0-30	QFT-GIT	19	10(53%)	43(33-48)	medium
Kiyoyasu Fukushima, 2021	Japan	0-30	QFT-GIT and T.SPOT.TB	118	64(54.2%)	39 (32-42)	low
Ji Young Hong, 2019	South Korea	31-100	QFT-GIT	27	22(81.5%)	42(35-46)	medium
Jin Takasaki, 2017	Japan	0-30	QFT-GIT and T.SPOT.TB	106	86(81.1%)	20(20-21)	low
Lina Yi, 2016	Japan	0-30	QFT-GIT	212	107(50.5%)	20(19-21)	low
Prof. Dr. Süheyla, 2020	Turkey	0-30	TST	30	17(56.7%)	NS	medium
Elif Böncüoğlu, MD, 2021	Turkey	0-30	TST	121	NS	NS	medium

IQR, interquartile range; QFT-GIT, QuantiFERON-TB Gold In-Tube; T.SPOT.TB, T-cell spot of tuberculosis assay; TST, tuberculin test

Table S17：Characteristics of the 31 studies included in the positive rates

First author, Year	Setting	TB burden of the areas	Comparator(s)	No. of participants	Females	Median age (IQR)	Risk of bias
H. Zhang, 2020	China	31-100	QFT-GIT	338	NS	NS	low
Meng-Rui Lee, 2019	Taiwan, China	31-100	QFT-GIT	223	111(49.8%)	NS	low
Mi Ra Ryu,2018	South Korea	31-100	QFT-GIT	317	112(35.3%)	53	low
Dongju Won, 2020	South Korea	31-100	QFT-GIT	220	97(44.1%)	47 (28-58)	medium
Thara K. Venkatappa, 2019	USA	0-30	QFT-GIT	508	250(49.2%)	32(19-44.5)	low
Yuzhen Xu, 2022	China	31-100	QFT-GIT	278	120(43.2%)	52(38-64)	low
Keita Takeda, 2020	Japan	0-30	QFT-GIT and T.SPOT.TB	35	11(31.4%)	58.6(22-86)	medium
Hee-Won Moon, 2017	USA	0-30	QFT-GIT	987	696(69.5%)	NS	low
Lucia Barcellini, 2016	Italy	0-30	QFT-GIT	119	56 (47.1%)	38	medium
Elia Noemi Gallegos Morales, 2017	Germany	0-30	QFT-GIT	134	72 (53.7%)	25.1	low
J.Knierrer, 2017	Germany	0-30	QFT-GIT	41	19(46.3%)	25.5	medium
Jung-Yien Chien, 2018	Taiwan, China	31-100	QFT-GIT	229	112(48.9%)	80	low
Soo Han Kim, 2020	South Korea	31-100	QFT-GIT	14	NS	NS	medium
Hee-Won Moon, 2020	South Korea	31-100	QFT-GIT	69	NS	35(27-50)	medium
Haoran Zhang, 2019	China	31-100	QFT-GIT and T.SPOT.TB	597	195(31.7%)	47(41-56)	high
Mizue Tsuyuzaki, 2019	Japan	0-30	QFT-GIT	412	192(46.6%)	44	low

Alexander W. Kay, 2019	USA	0-30	QFT-GIT	46	27(59%)	NS	high
Keita Takeda, 2020	Japan	0-30	QFT-GIT	50	45(90%)	29.3 (21-45)	medium
Hidetoshi Igari, 2021	Japan	0-30	T.SPOT.TB	184	24(13%)	49(40-58)	medium
Hidetoshi Igari, 2017	Japan	0-30	T.SPOT.TB	154	126(81.8%)	66.5	medium
Hidetoshi Igari, 2019	Japan	0-30	T.SPOT.TB	136	55(40.4%)	49	medium
Ana Fernández-Blázquez, 2021	Japan	0-30	T.SPOT.TB	1464	728(49.7%)	NS	medium
Cory Primaturia, 2020	Indonesia	201-	TST	71	37(52.1%)	11.8	medium
Suchitra Surve, 2021	India	101-200	TST	123	NS	3	medium
Kirtilaxmi Benachinmard, 2021	India	101-200	TST	77	47(61%)	NS	medium
Fatima Nawaf Abdulkareem, 2020	India	101-200	TST	309	NS	NS	medium
Nuntana Chumpa, 2022	Thailand	101-200	TST	158	74(46.8%)	22	low
Sivaporn Gatechompol, 2021	Thailand	101-200	TST	294	0(0%)	38(32-50)	low
Prof. Dr. Süheyla, 2020	Turkey	0-30	TST	92	40(43.5%)	41.8(18-72)	medium
Violet N.Chihota, 2022	South African	201-	TST	235	4(1.7%)	48 (44-53)	medium
Elif Böncüoğlu, MD, 2021	Turkey	0-30	TST	99	NS	NS	medium
U. Gurjav, 2019	Mongolia	201-	TST	285	151(53.0%)	NS	high

IQR, interquartile range; QFT-GIT, QuantiFERON-TB Gold In-Tube; T.SPOT.TB, T-cell spot of tuberculosis assay; TST, tuberculin test

Table S18：Linearregression test of funnel plot asymmetry results of QFT-PLUS compared to QFT-GIT, T-SPOT.TB and TST in three populations.

	**patients with active TB**			**populations with very low risk of TB exposure**			**high-risk populations**		
	**t**	**df**	**p-value**	**t**	**df**	**p-value**	**t**	**df**	**p-value**
QFT-GIT	0.12	9	0.9100	NA	NA	NA	-1.37	16	0.1905
T-SPOT.TB	NA	NA	NA	NA	NA	NA	NA	NA	NA
TST	NA	NA	NA	NA	NA	NA	-1.24	8	0.2516

Figure S1: Forest plot of studies estimating the sensitivity of QFT-Plus (A) and QFT-GIT (B) in patients with active tuberculosis

A. QFT-Plus

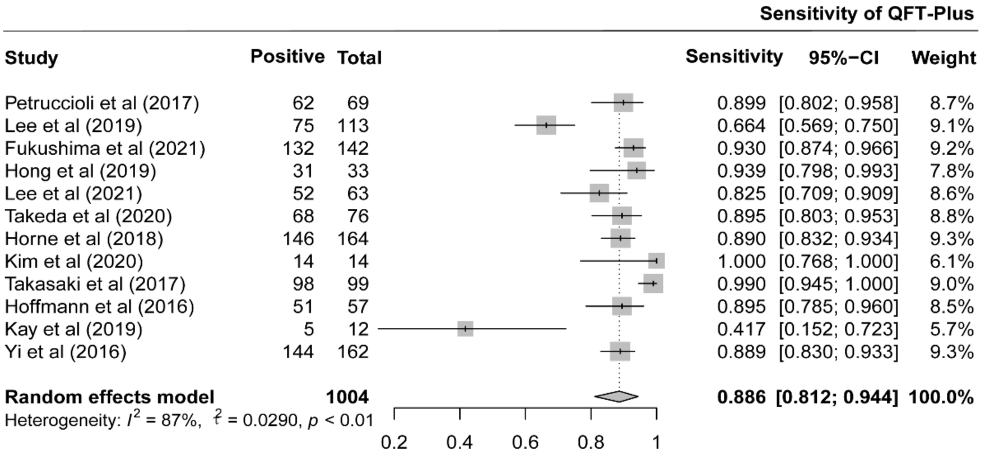

B. QFT-GIT

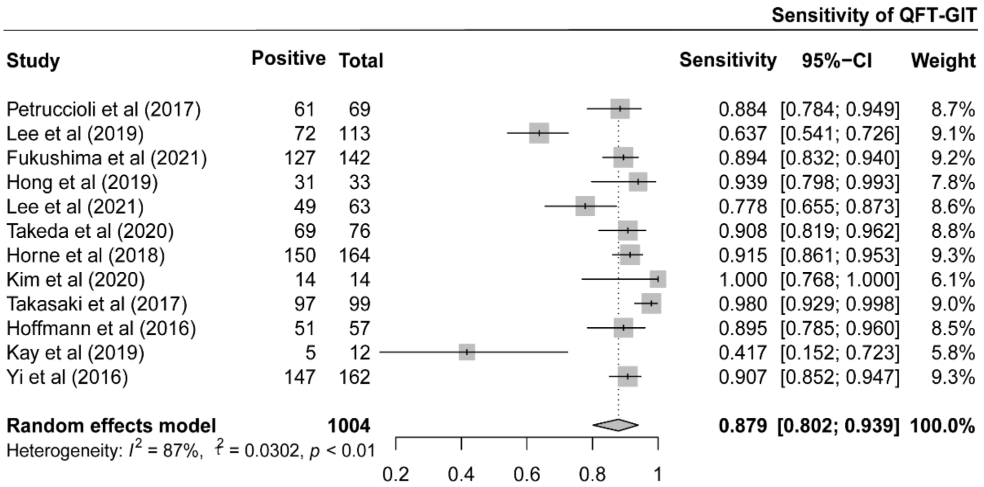

Figure S2：Forest plot of studies estimating the sensitivity of QFT-Plus (A) and T-SPOT.TB (B) in patients with active tuberculosis

A. QFT-Plus

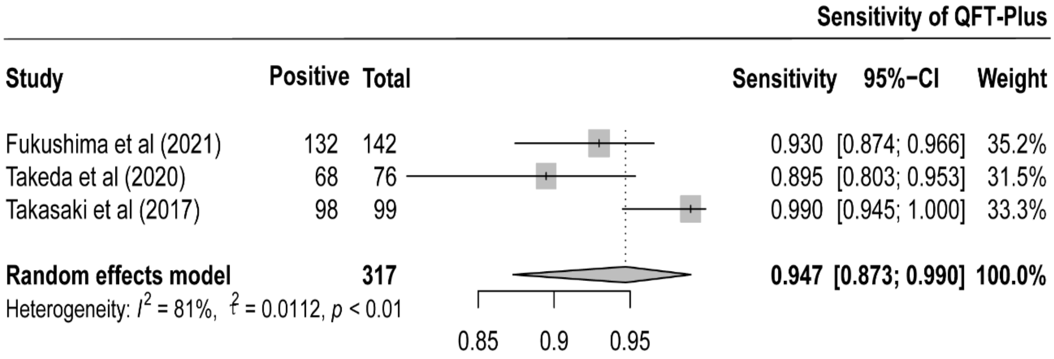

B. T-SPOT.TB

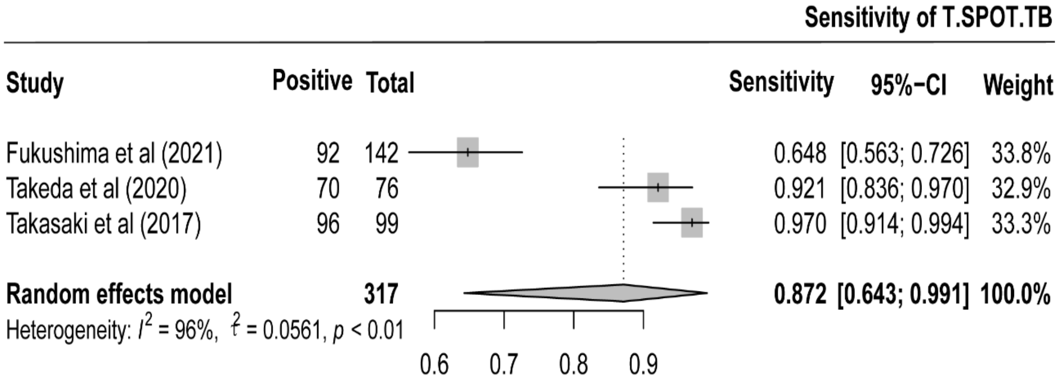

Figure S3：Forest plot of studies estimating the specificity of QFT-Plus (A) and QFT-GIT (B) in populations with very low risk of TB exposure.

A. QFT-Plus

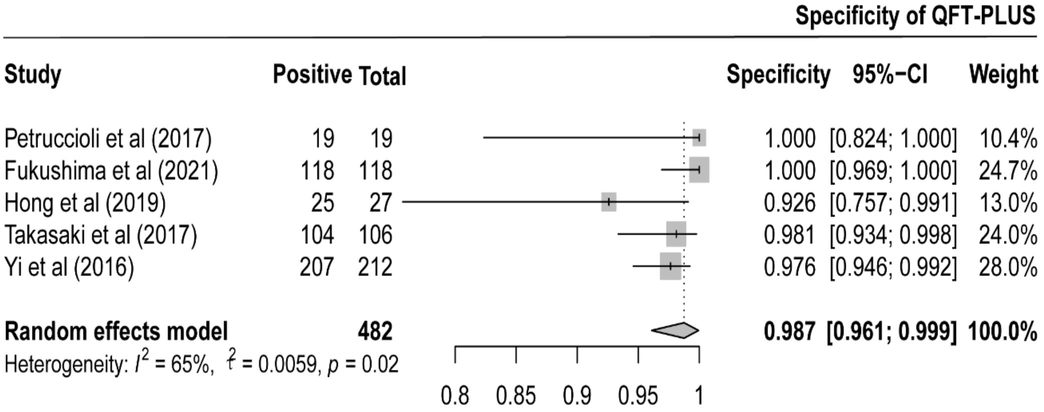

B. QFT-GIT

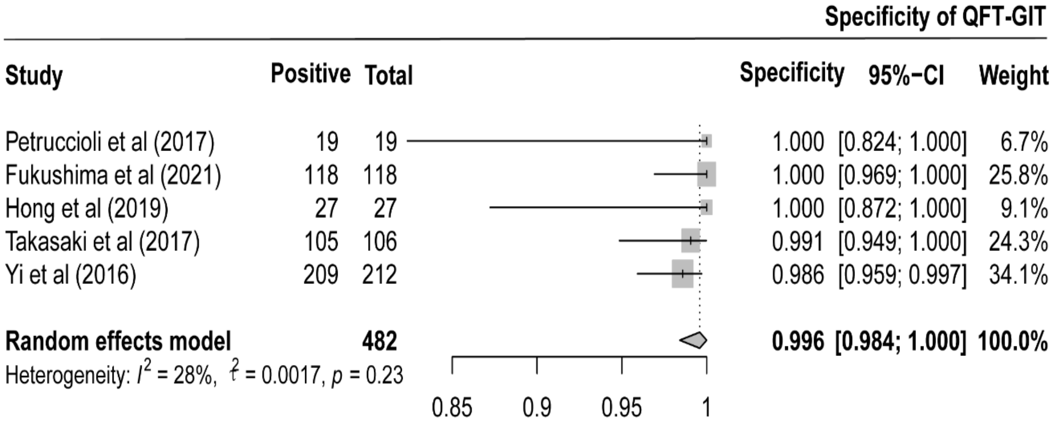

Figure S4: Forest plot of studies estimating the specificity of QFT-Plus (A) and T-SPOT.TB (B) in populations with very low risk of TB exposure.

A. QFT-Plus

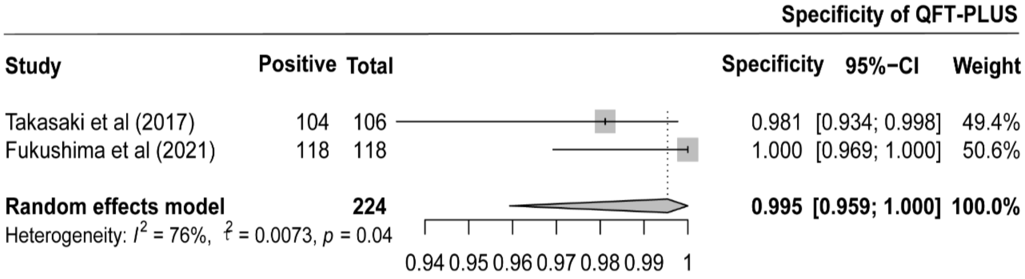

B. T-SPOT.TB

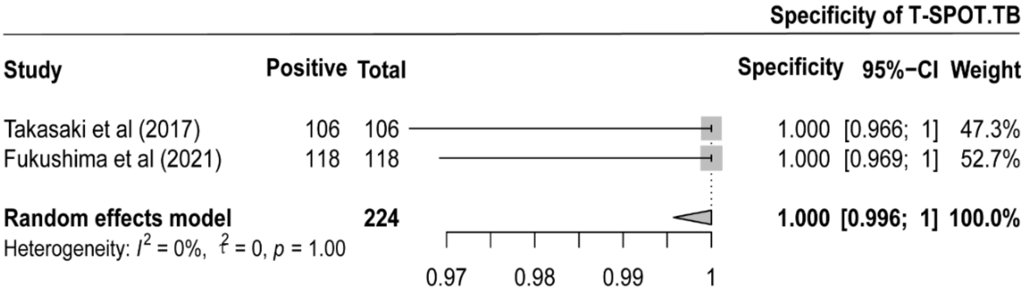

Figure S5：Forest plot of studies estimating the specificity of QFT-Plus (A) and TST (B) in populations with very low risk of TB exposure.

A. QFT-Plus

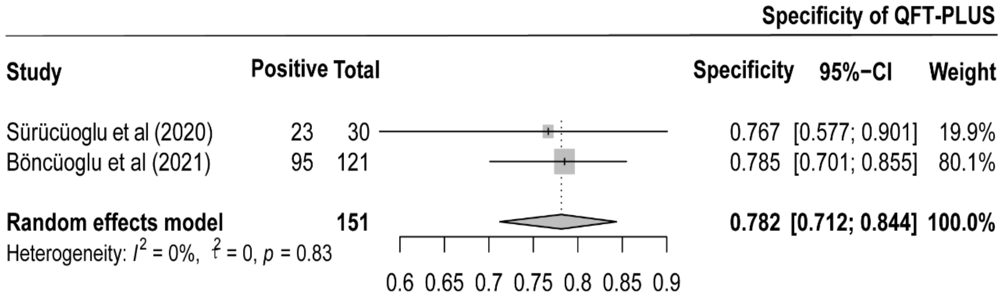

B. TST

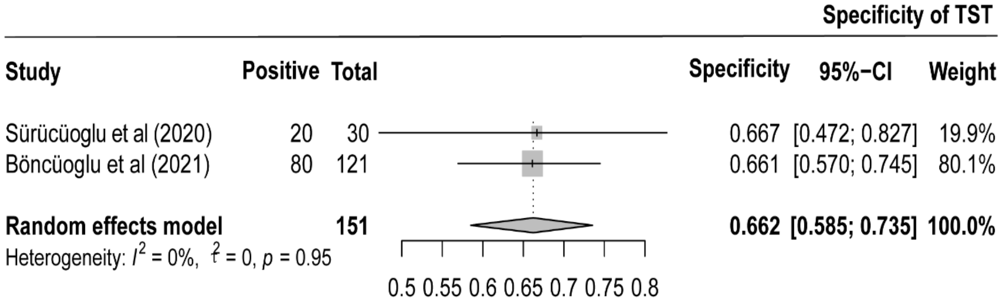

Figure S6：Forest plot of studies estimating the positive rate Plus (A) and QFT-GIT (B) in high-risk populations.

A. QFT-Plus

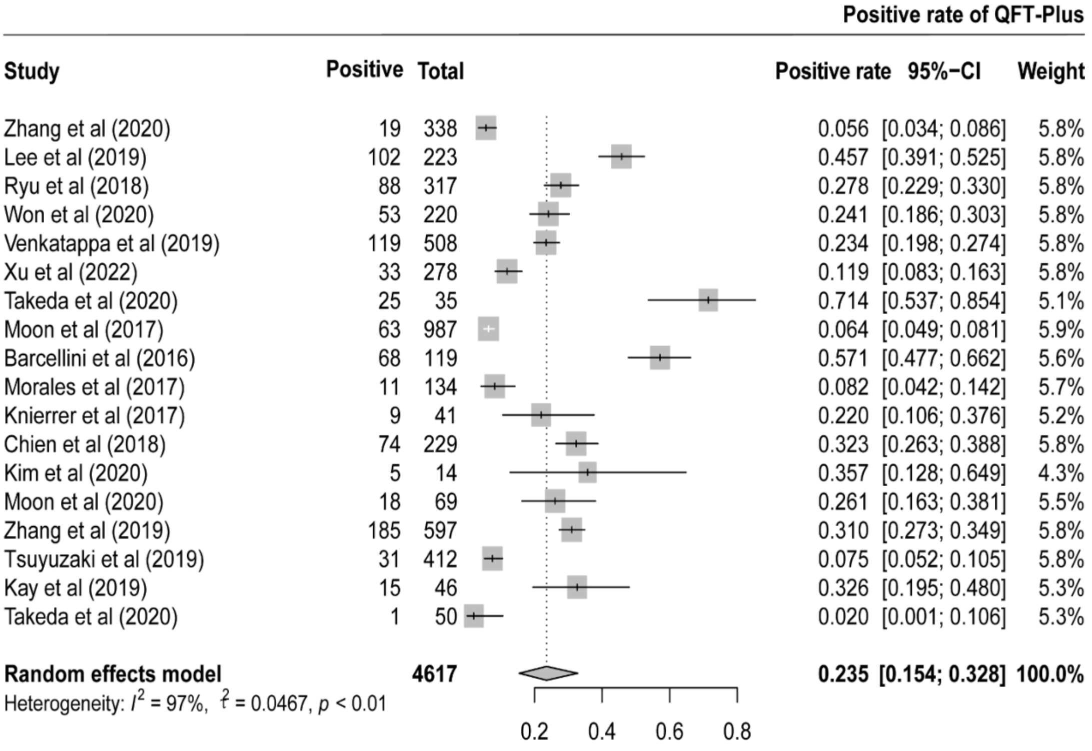

B. QFT-GIT

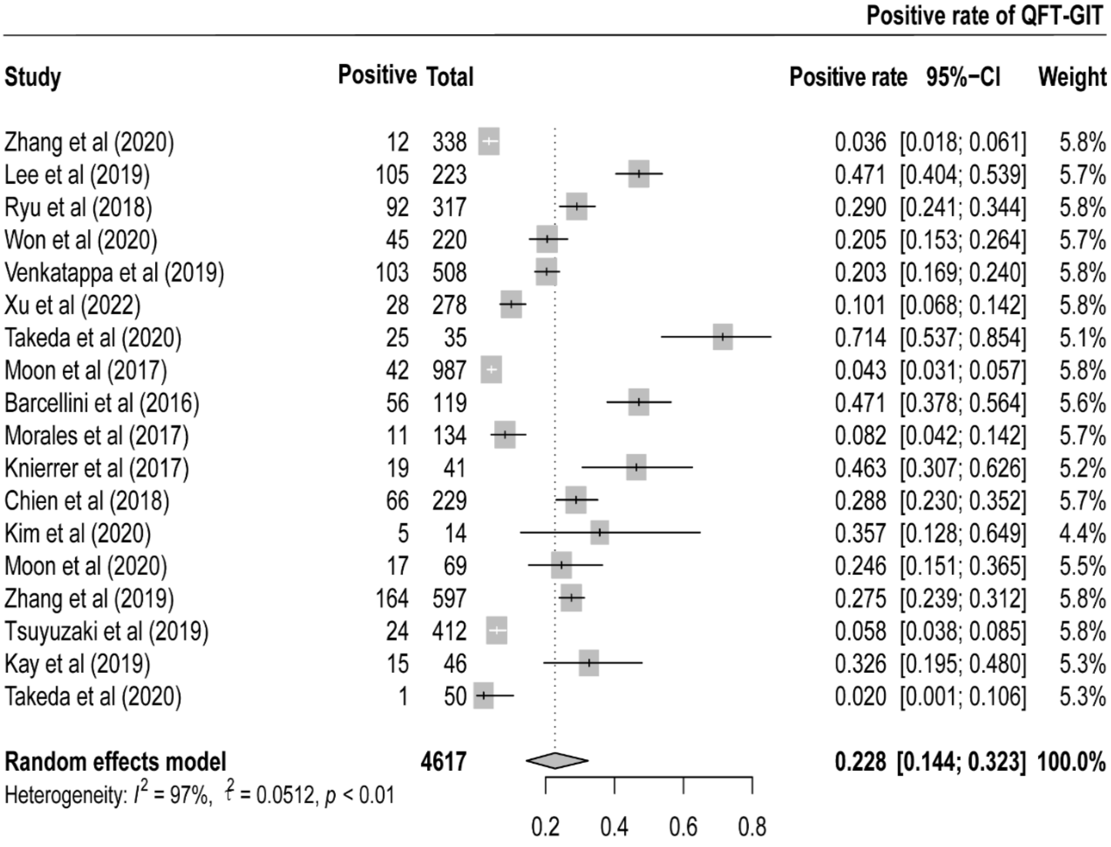

Figure S7：Forest plot of studies estimating the positive rate of QFT-Plus (A) and T-SPOT.TB (B) in high-risk populations.

A. QFT-Plus

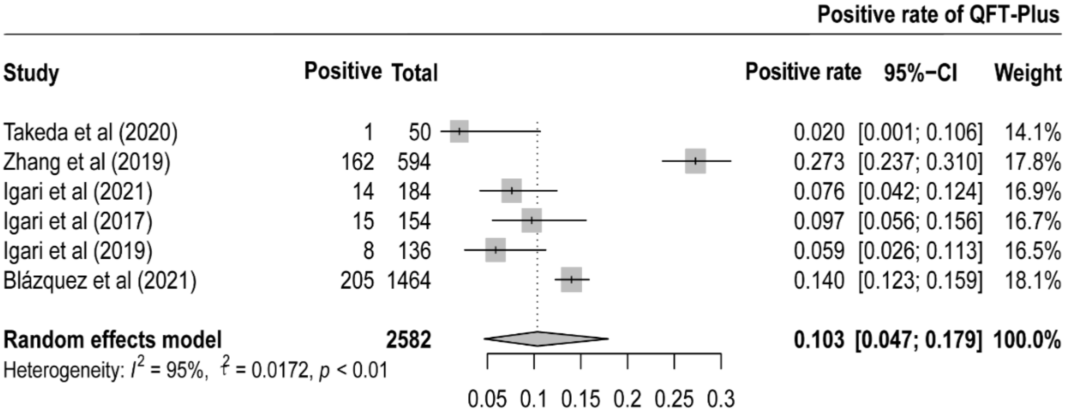

B. T-SPOT.TB

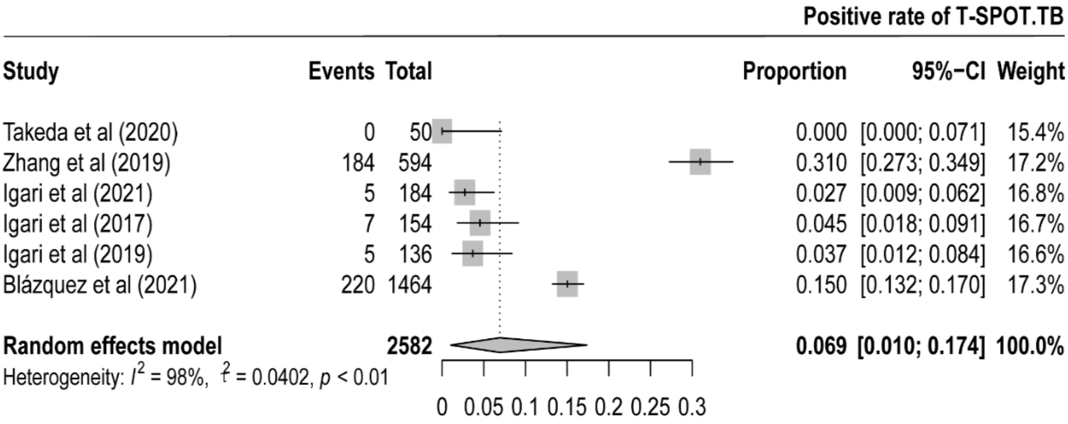

Figure S8：Forest plot of studies estimating the positive rate of QFT-Plus (A) and TST (B) in high-risk populations.

A. QFT-Plus

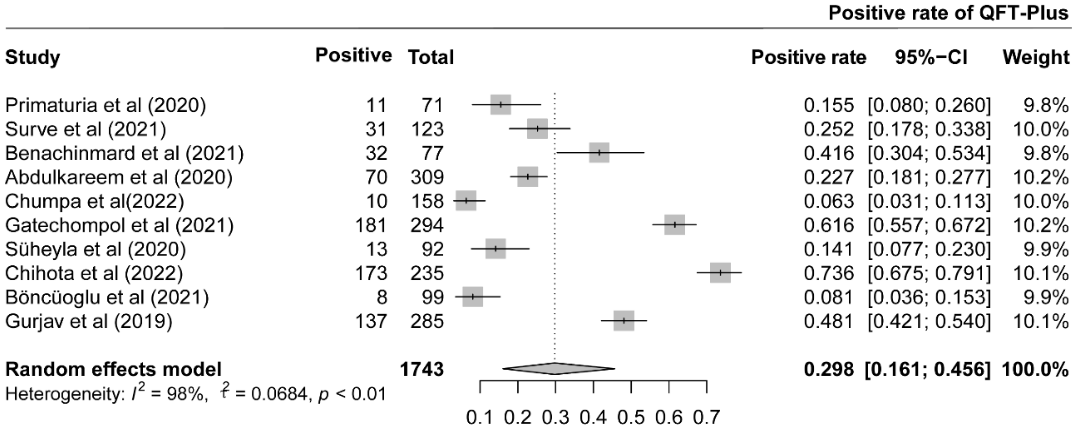

B. TST

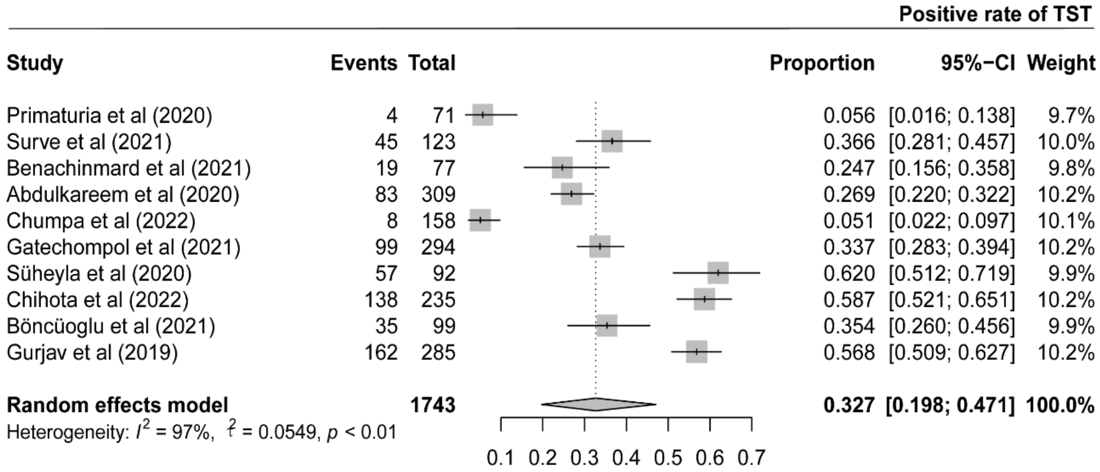

Figure S9：Forest plot of studies estimating the sensitivity in patients with active tuberculosis for age of the participants (A), TB burden of the areas (B) and number of participants (C) subgroup analysis of QFT-PLUS compared with QFT-GIT.

A. Age of the participants

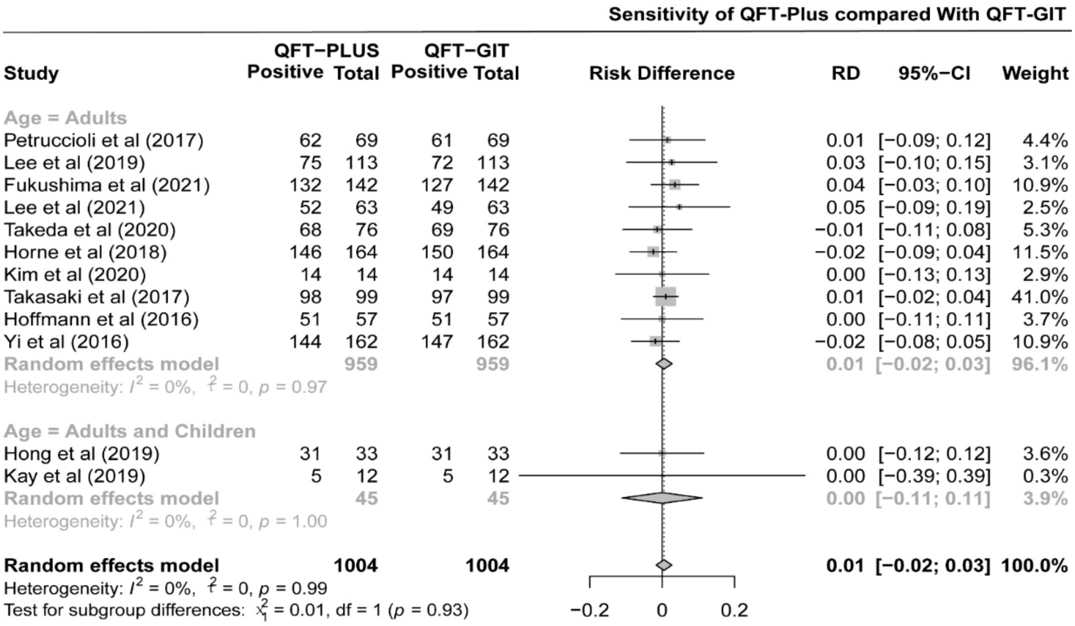

B. TB burden of the areas

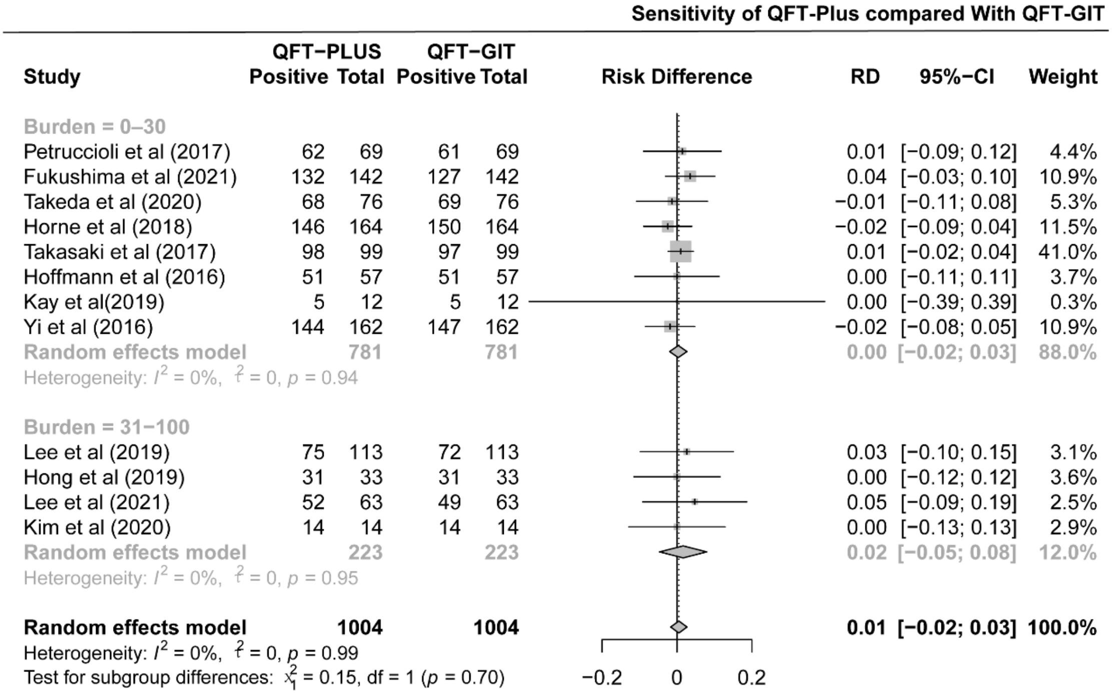

C. Number of participants

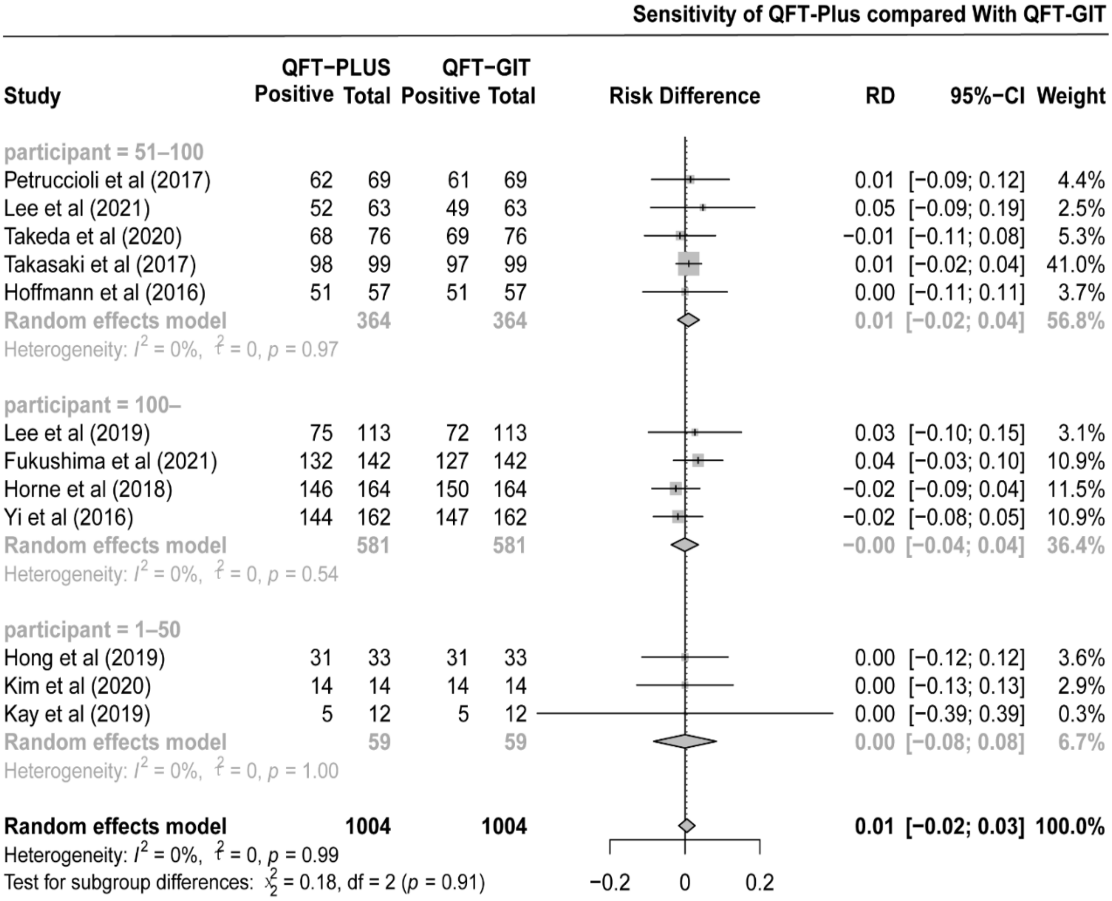

Figure S10：Forest plot of studies estimating the sensitivity in patients with active tuberculosis for number of participants subgroup analysis of QFT-PLUS compared with T-SPOT.TB.

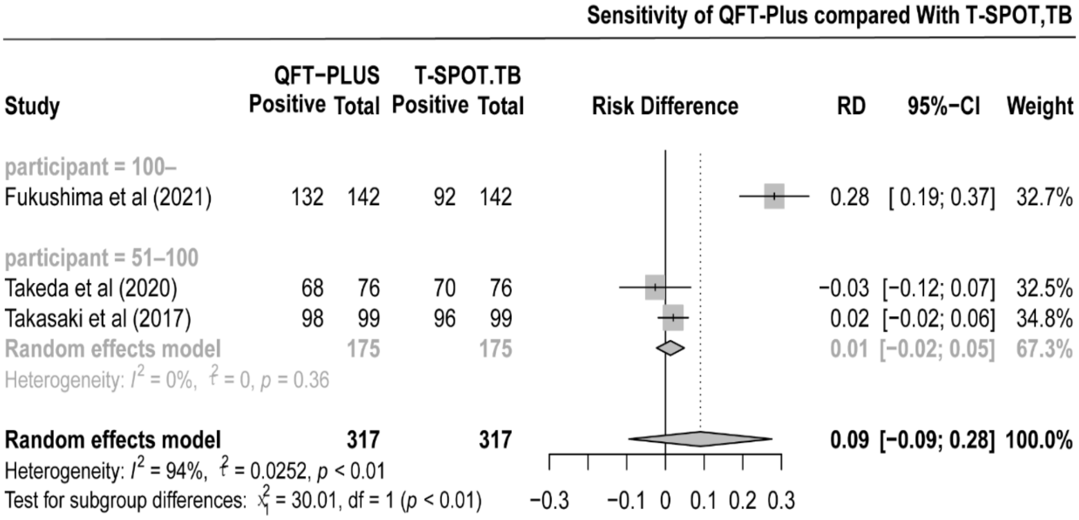

Figure S11：Forest plot of studies estimating the Specificity in populations with very low risk of TB exposure for TB burden of the areas (A) and number of participants (B) subgroup analysis of QFT-PLUS compared with QFT-GIT.

A. TB burden of the areas

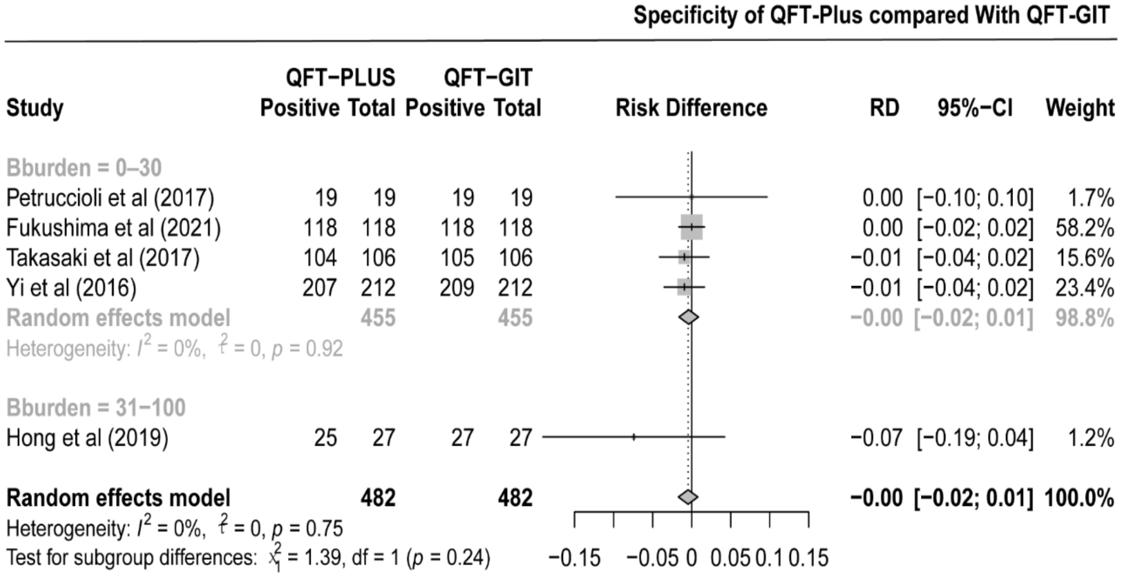

B. Number of participants

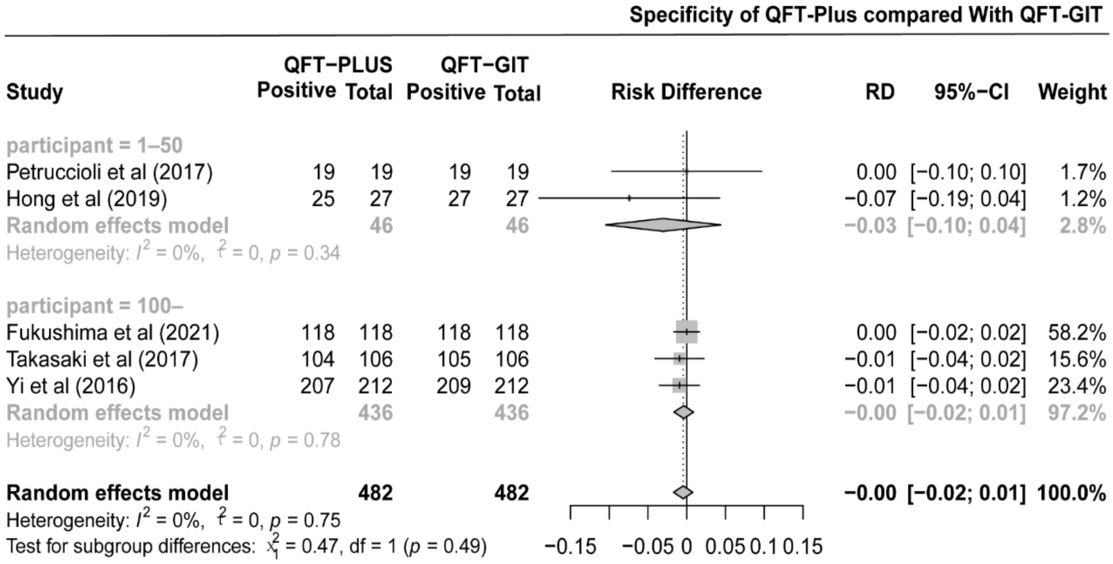

Figure S12：Forest plot of studies estimating the positive rate in high-risk populations for age of the participants (A), TB burden of the areas (B), number of participants (C) and population (D)subgroup analysis of QFT-PLUS compared with QFT-GIT.

A. Age of the participants

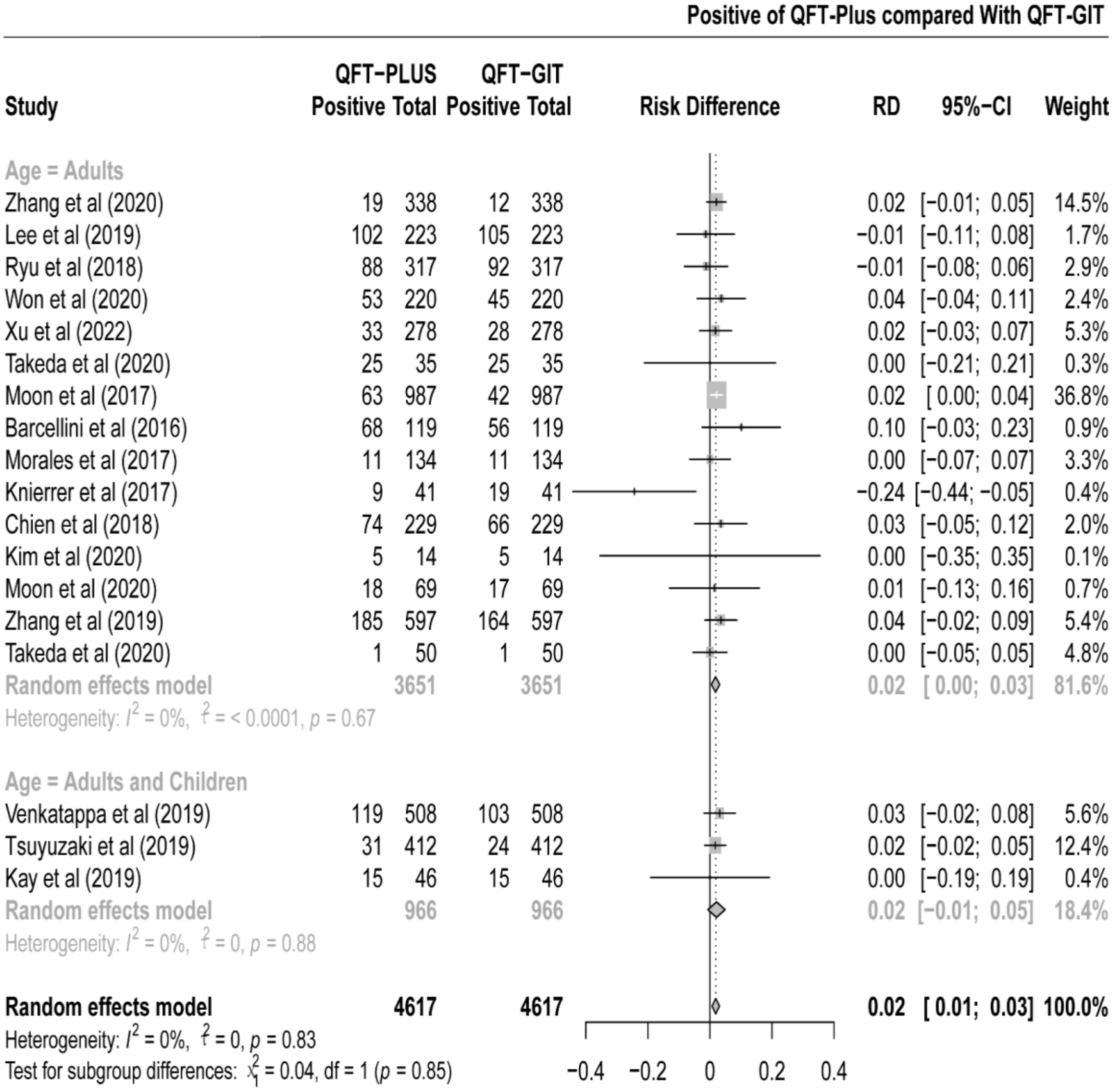

B. TB burden of the areas

Positive rate of QFT-Plus compared With QFT-GIT

Study	QFT-PLUS Positive	QFT-PLUS Total	QFT-GIT Positive	QFT-GIT Total	RD	95%-CI	Weight
**Burden = 31–100**							
Zhang et al (2020)	19	338	12	338	0.02	[−0.01; 0.05]	14.5%
Lee et al (2019)	102	223	105	223	−0.01	[−0.11; 0.08]	1.7%
Ryu et al (2018)	88	317	92	317	−0.01	[−0.08; 0.06]	2.9%
Won et al (2020)	53	220	45	220	0.04	[−0.04; 0.11]	2.4%
Xu et al (2022)	33	278	28	278	0.02	[−0.03; 0.07]	5.3%
Chien et al (2018)	74	229	66	229	0.03	[−0.05; 0.12]	2.0%
Kim et al (2020)	5	14	5	14	0.00	[−0.35; 0.35]	0.1%
Moon et al (2020)	18	69	17	69	0.01	[−0.13; 0.16]	0.7%
Zhang et al (2019)	185	597	164	597	0.04	[−0.02; 0.09]	5.4%
**Random effects model**		**2285**		**2285**	**0.02**	**[0.00; 0.04]**	**35.1%**
Heterogeneity: $I^2 = 0\%$, $\tau^2 = 0$, $p = 0.98$							
**Burden = 0–30**							
Venkatappa et al (2019)	119	508	103	508	0.03	[−0.02; 0.08]	5.6%
Takeda et al (2020)	25	35	25	35	0.00	[−0.21; 0.21]	0.3%
Moon et al (2017)	63	987	42	987	0.02	[0.00; 0.04]	36.8%
Barcellini et al (2016)	68	119	56	119	0.10	[−0.03; 0.23]	0.9%
Morales et al (2017)	11	134	11	134	0.00	[−0.07; 0.07]	3.3%
Knierrer et al (2017)	9	41	19	41	−0.24	[−0.44; −0.05]	0.4%
Tsuyuzaki et al (2019)	31	412	24	412	0.02	[−0.02; 0.05]	12.4%
Kay et al (2019)	15	46	15	46	0.00	[−0.19; 0.19]	0.4%
Takeda et al (2020)	1	50	1	50	0.00	[−0.05; 0.05]	4.8%
**Random effects model**		**2332**		**2332**	**0.02**	**[0.00; 0.03]**	**64.9%**
Heterogeneity: $I^2 = 16\%$, $\tau^2 = < 0.0001$, $p = 0.30$							
**Random effects model**		**4617**		**4617**	**0.02**	**[0.01; 0.03]**	**100.0%**
Heterogeneity: $I^2 = 0\%$, $\tau^2 = 0$, $p = 0.83$							
Test for subgroup differences: $\chi^2_1 = 0.02$, df = 1 ($p = 0.89$)							

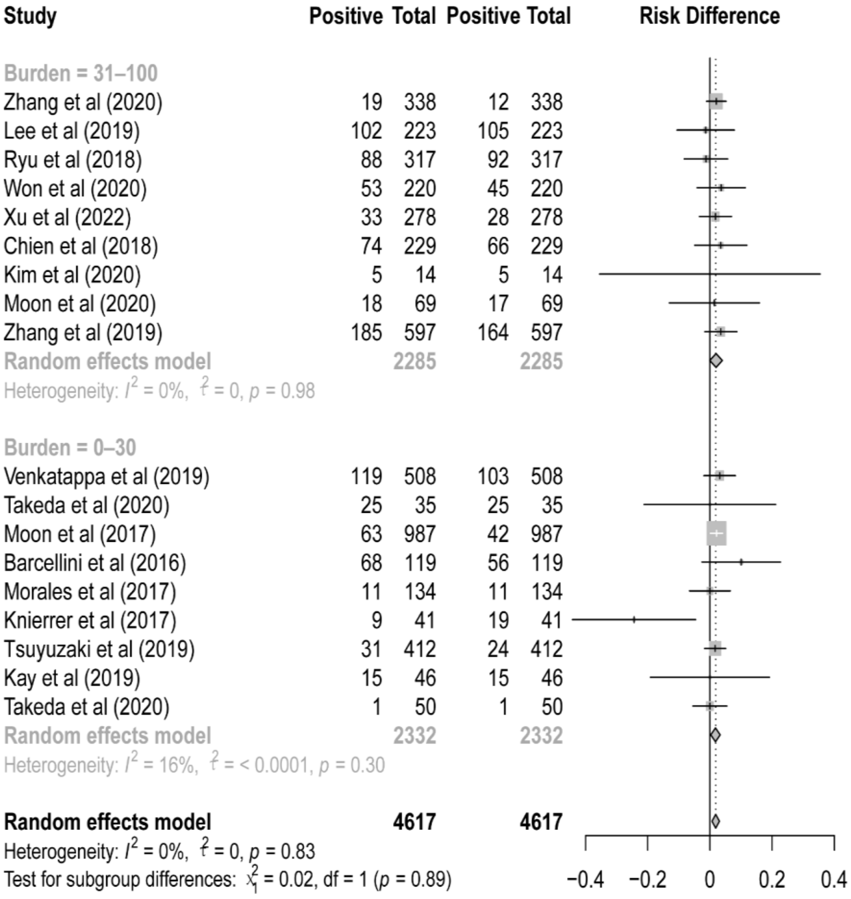

C. Number of participants

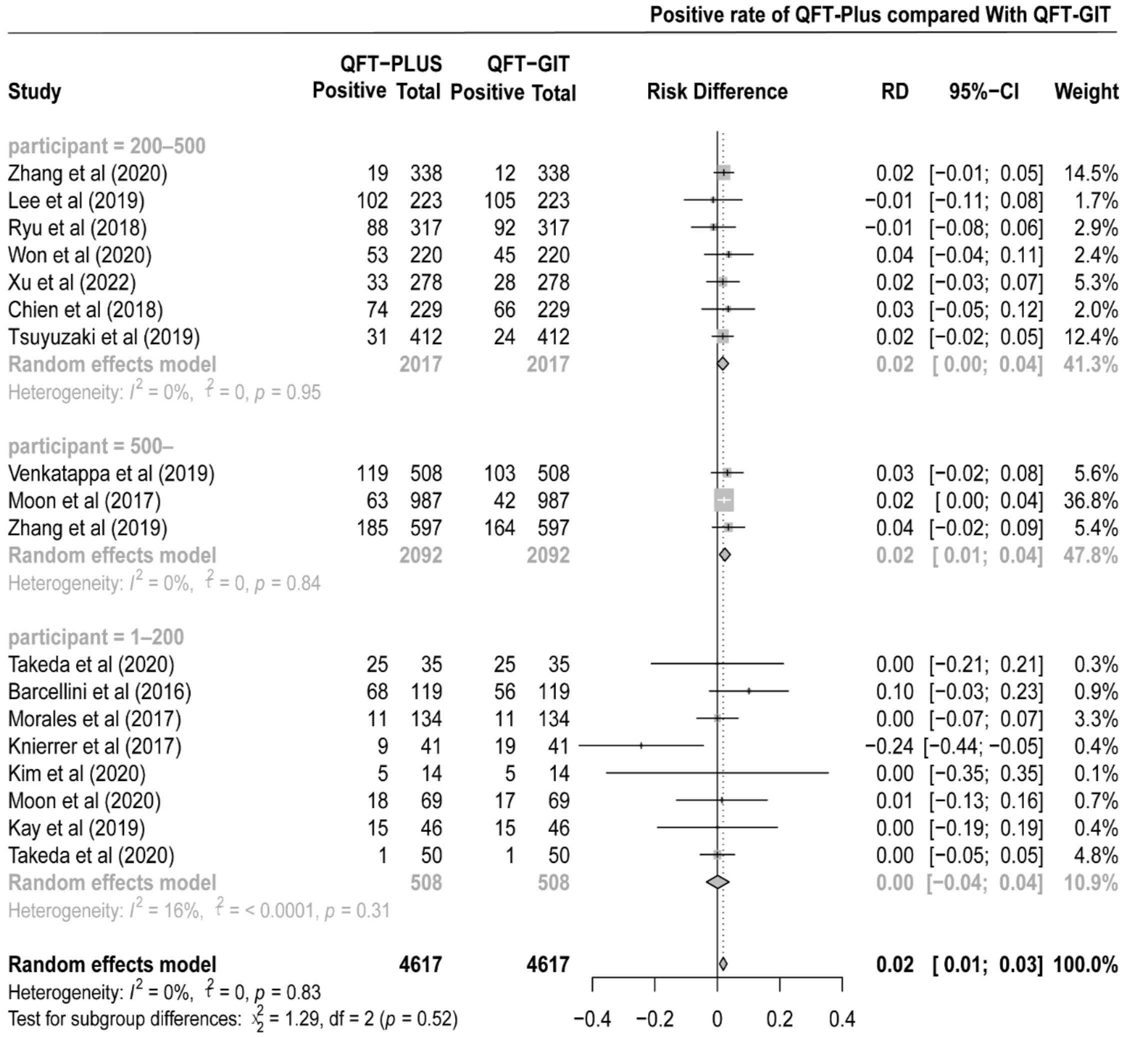

D. Population

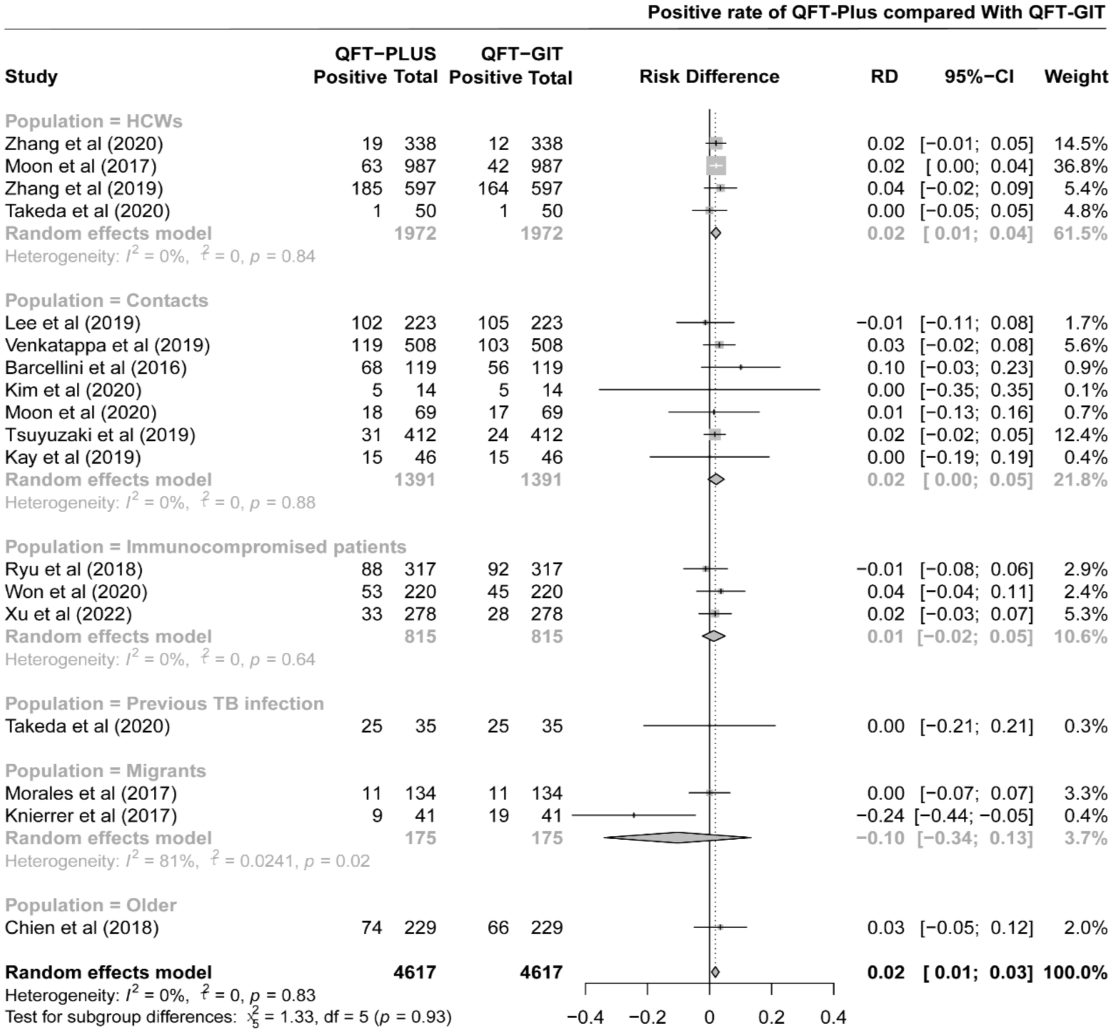

Figure S13：Forest plot of studies estimating the positive rate in high-risk populations for age of the participants (A), TB burden of the areas (B), number of participants (C) and population (D)subgroup analysis of QFT-PLUS compared with T-SPOT.TB.

A. Age of the participants

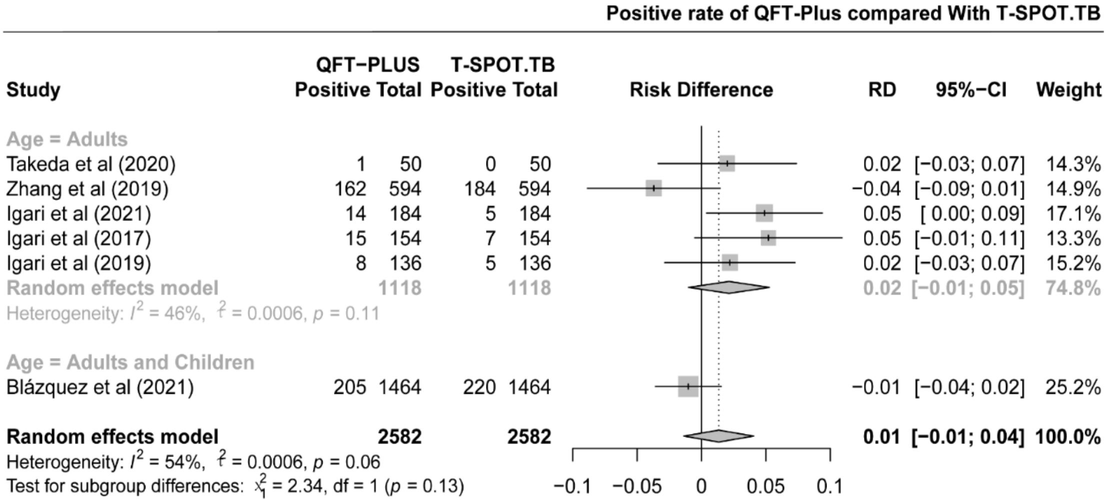

B. TB burden of the areas

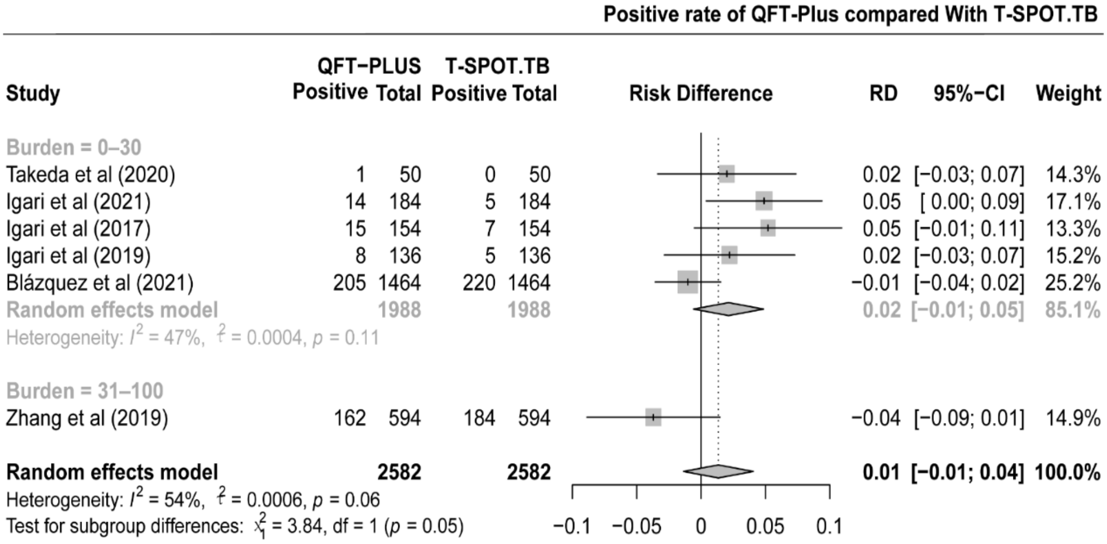

C. Number of participants

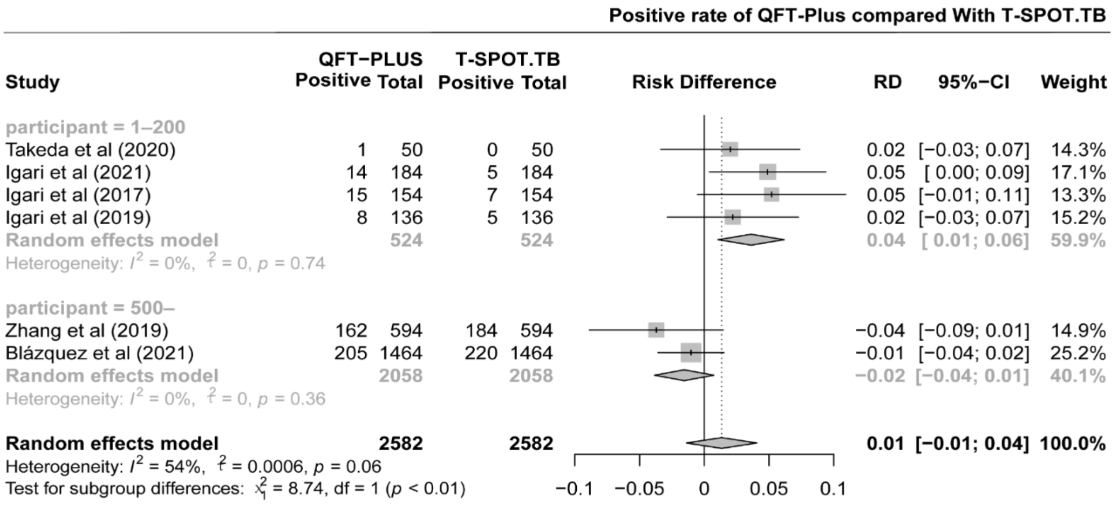

D. Population

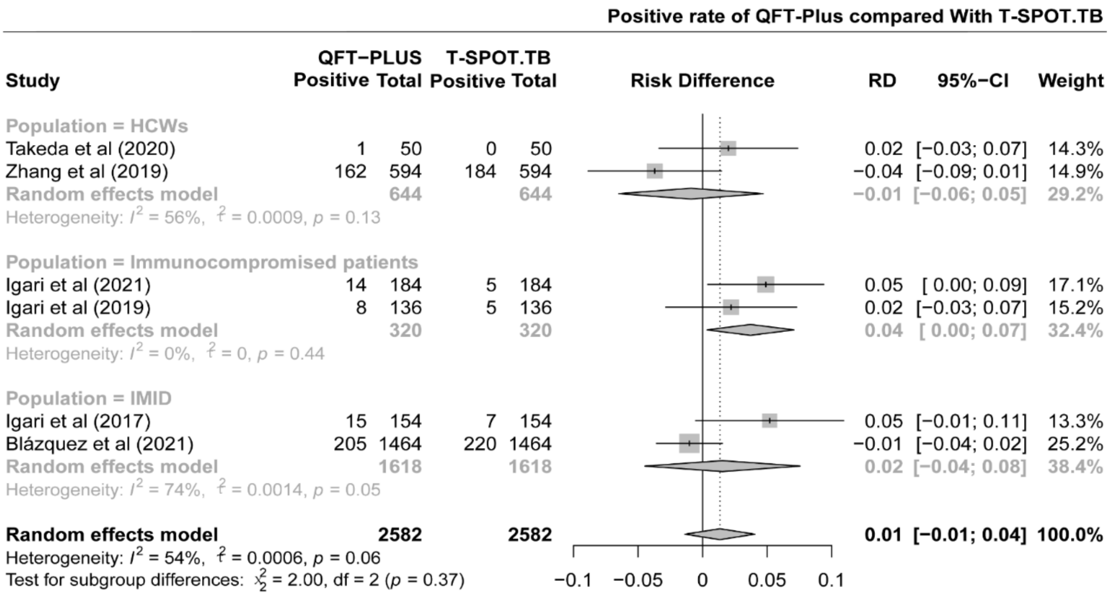

Figure S14：Forest plot of studies estimating the positive rate in high-risk populations for age of the participants (A), TB burden of the areas (B), number of participants (C) and population (D)subgroup analysis of QFT-PLUS compared with TST.

A. Age of the participants

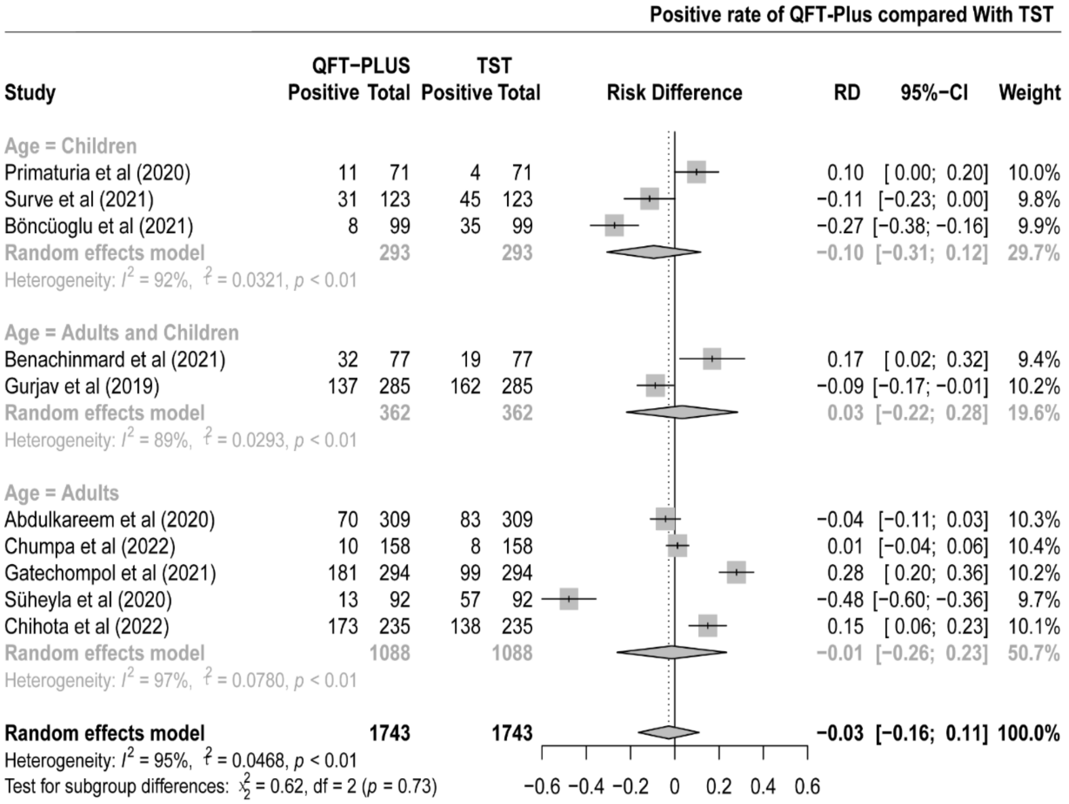

B. TB burden of the areas

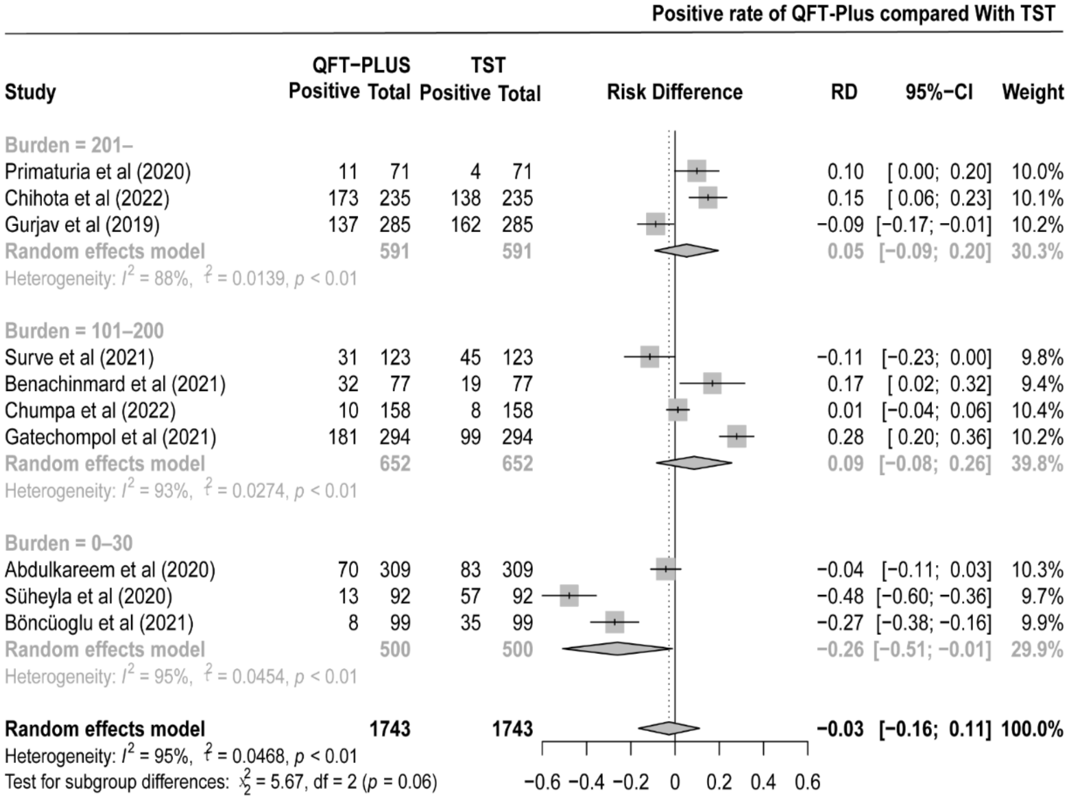

C. Number of participants

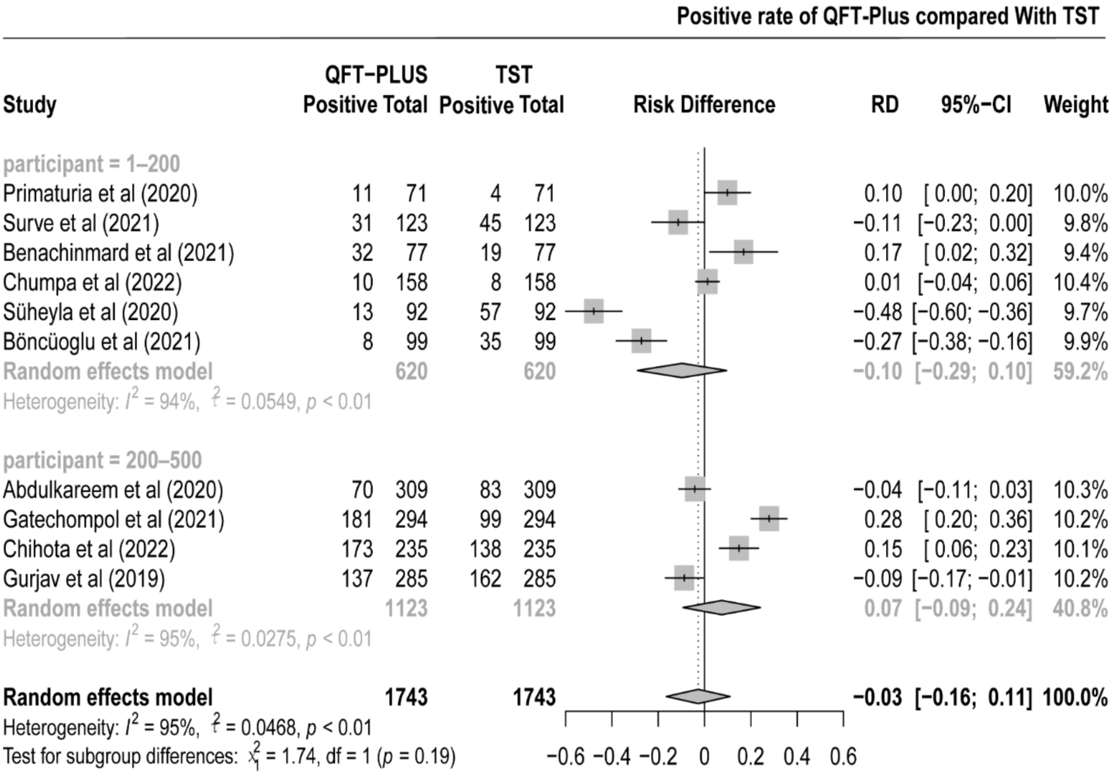

D. Population

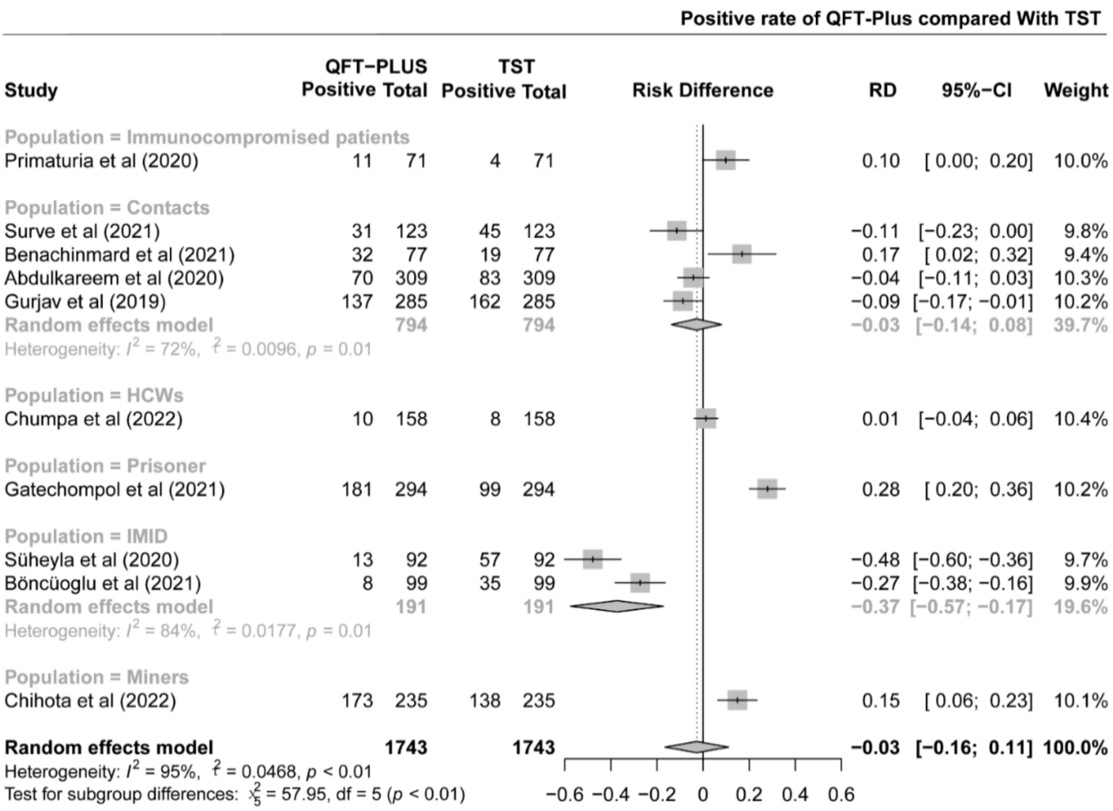

Figure S15：Sensitivity analysis of QFT-PLUS compared to QFT-GIT (A) and T-SPOT.TB (B) in patients with active TB

A. QFT-GIT

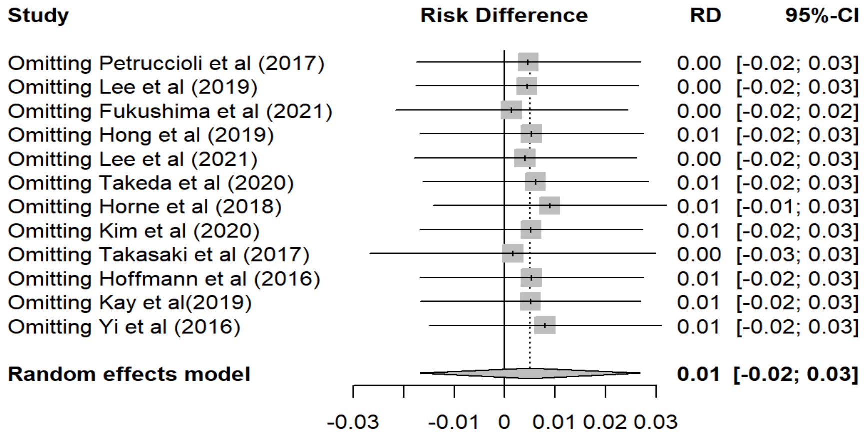

B. T-SPOT.TB

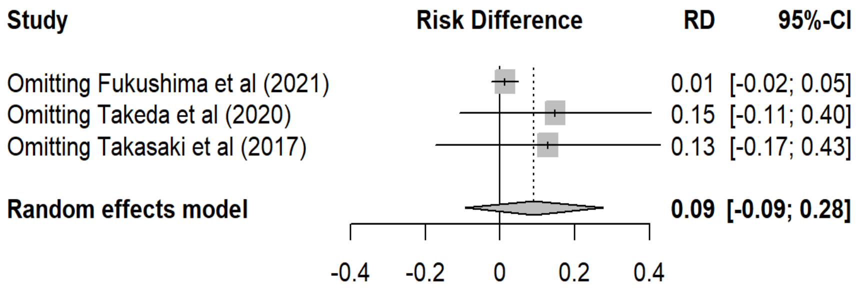

Figure S16: Sensitivity analysis of QFT-PLUS compared to QFT-GIT (A), T-SPOT.TB (B) and TST(C) in populations with very low risk of TB exposure

A. QFT-GIT

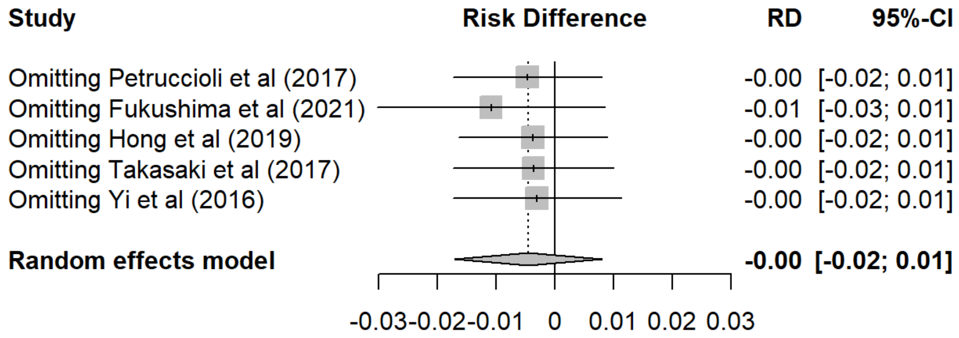

B. T-SPOT.TB

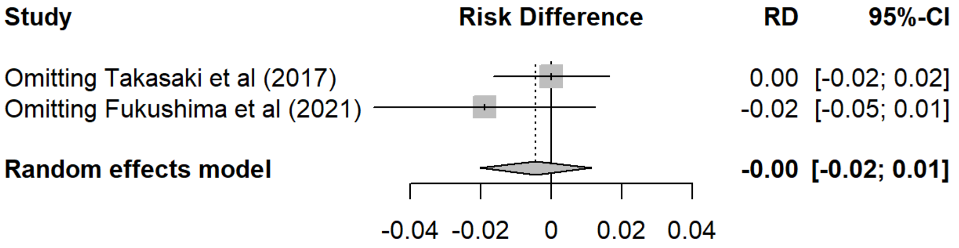

C. TST

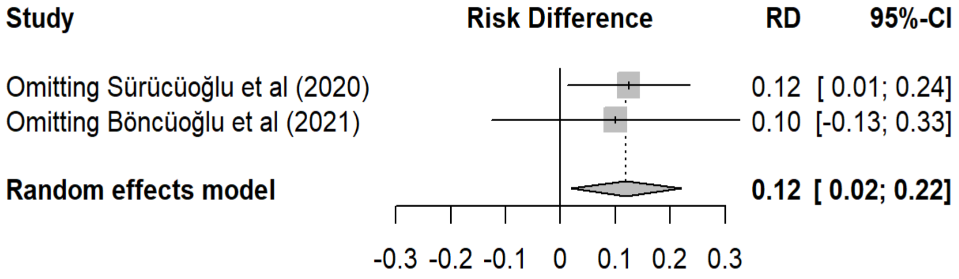

Figure S17: Sensitivity analysis of QFT-PLUS compared to QFT-GIT (A), T-SPOT.TB (B) and TST(C) in high-risk populations

A. QFT-GIT

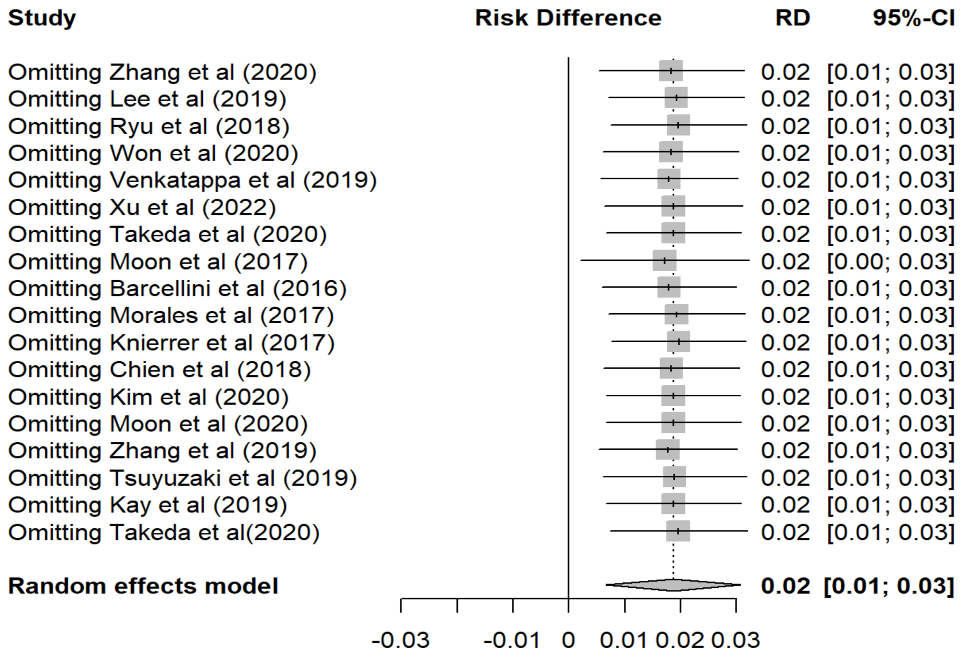

B. T-SPOT.TB

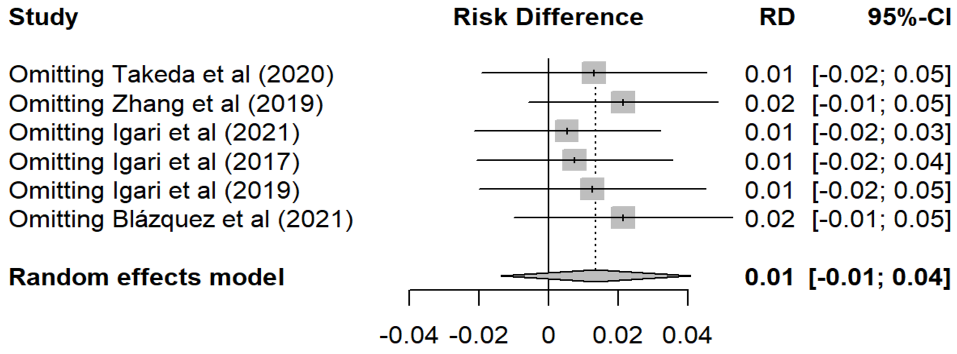

C. TST

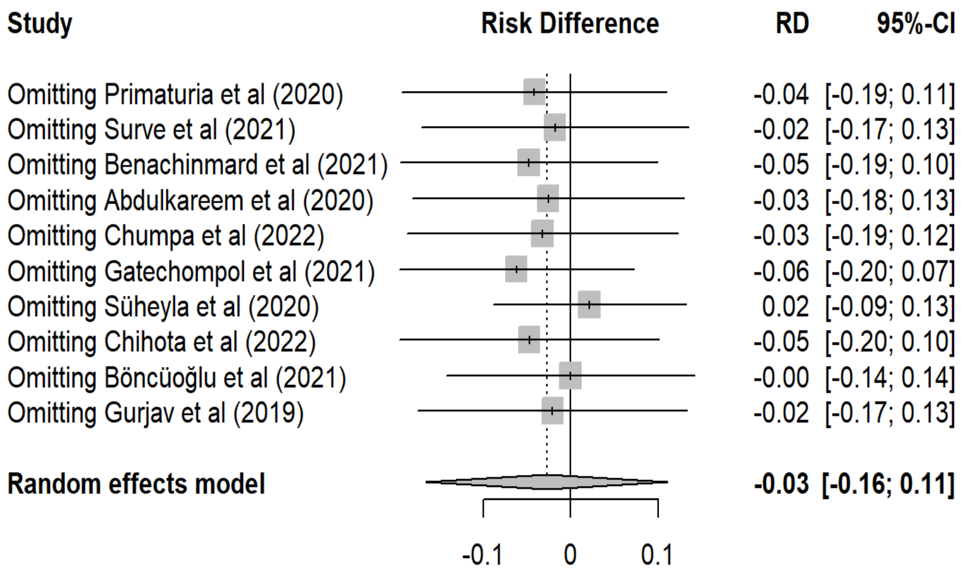

Figure S18：Funnel plot of QFT-PLUS compared to QFT-GIT in patients with active TB

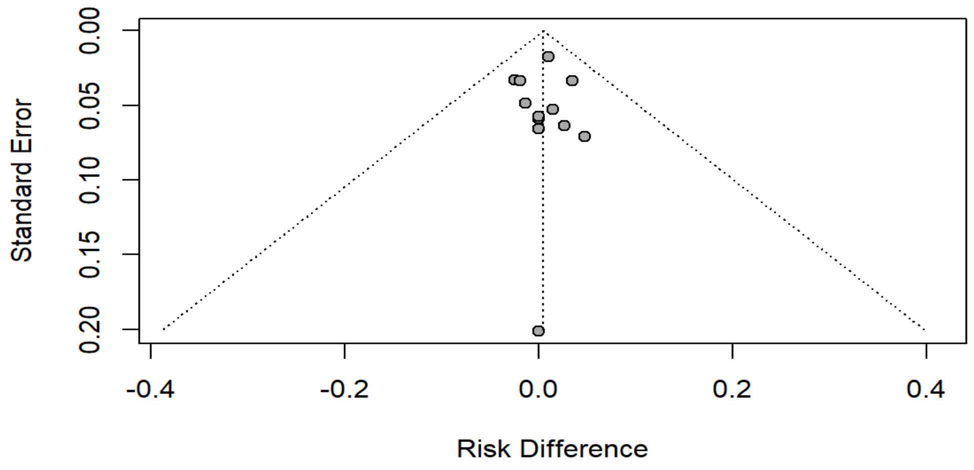

Figure S19：Funnel plot of QFT-PLUS compared to QFT-GIT (A) and TST (B) in high-risk populations

A. QFT-GIT

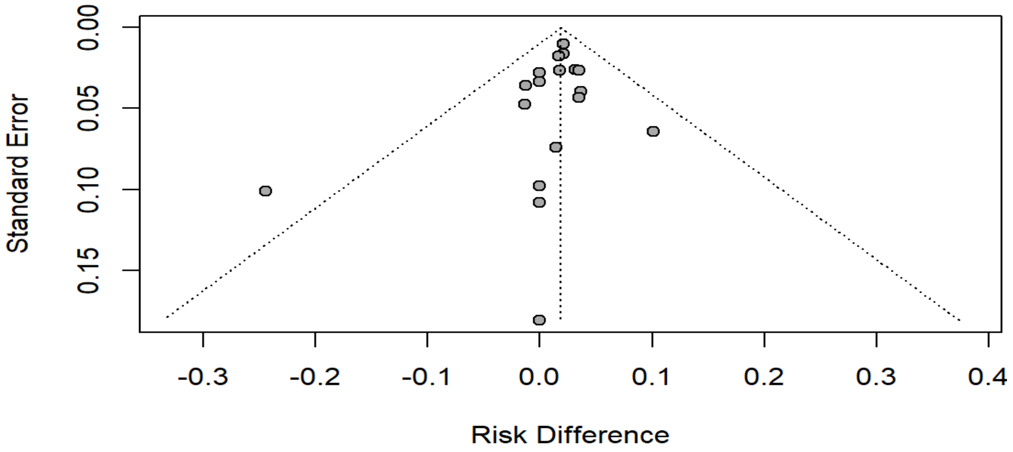

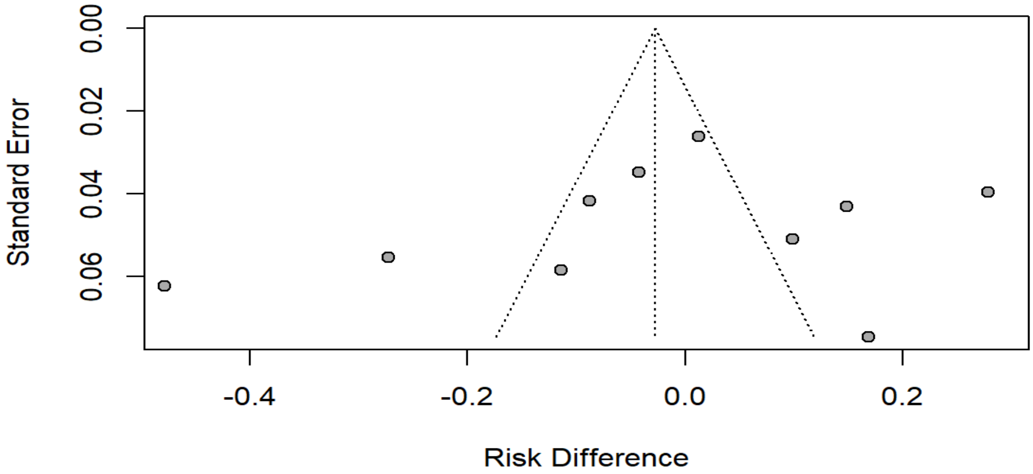

Supplement: Supplementary file 1 — Additional file 1: Table S1. PRISMA checklist. Table S2. Search strategy. Table S2. Search strategy. Table S3. Inclusion and partial exclusion for patients with active TB. Table S4. Inclusion and partial exclusion for populations with very low risk of TB exposure. Table S5. Inclusion and partial exclusion for high-risk populations. Table S6. Populations considered high-risk. Table S7. Details of excluded criteria. Table S8. QUADAS-2 adapted quality assessment criteria for patients with active TB. Table S9. Quality score of 12 studies for patients with active TB. Table S10. QUADAS-2 adapted quality assessment criteria for populations with very low risk of TB exposure. Table S11. Quality score of 7 studies for populations with very low risk of TB exposure. Table S12. QUADAS-2 adapted quality assessment criteria for high-risk groups. Table S13. Quality score of 31 studies for high-risk groups. Table S14. Reasons for exclusion of 42 studies that were read in full-text review. Table S15. Characteristics of the 12 studies included in the sensitivity analysis. Table S16. Characteristics of the 7 studies included in the specificity analysis. Table S17. Characteristics of the 31 studies included in the positive rates. Table S18. Linearregression test of funnel plot asymmetry results of QFT-PLUS compared to QFT-GIT, T-SPOT.TB and TST in three populations. Figure S1. Forest plot of studies estimating the sensitivity of QFT-Plus (A) and QFT-GIT (B) in patients with active tuberculosis. Figure S2. Forest plot of studies estimating the sensitivity of QFT-Plus (A) and T-SPOT.TB (B) in patients with active tuberculosis. Figure S3. Forest plot of studies estimating the specificity of QFT-Plus (A) and QFT-GIT (B) in populations with very low risk of TB exposure. Figure S4. Forest plot of studies estimating the specificity of QFT-Plus (A) and T-SPOT.TB (B) in populations with very low risk of TB exposure. Figure S5. Forest plot of studies estimating the specificity of QFT-Plus (A) and [file 12879_2023_8008_MOESM1_ESM.pdf]
